# Test accuracy of glomerular filtration rate estimation with creatinine and cystatin C in adults with moderate chronic kidney disease: prospective cohort study

**DOI:** 10.1136/bmjmed-2025-001827

**Published:** 2026-01-21

**Authors:** Edmund J Lamb, Jonathan Barratt, Elizabeth Ann Brettell, Paul Cockwell, R Neil Dalton, Jonathan James Deeks, Gillian Eaglestone, Philip Kalra, Kamlesh Khunti, Fiona C Loud, Phoebe Amelie Mead, Ryan Ottridge, Tracy Pellatt-Higgins, Aisling Potter, Ceri Rowe, Katie Scandrett, Claire Sharpe, Bethany Shinkins, Alice J Sitch, Alison Smith, Paul E Stevens, Andrew J Sutton, Maarten Taal

**Affiliations:** 1 Clinical Biochemistry, East Kent Hospitals University NHS Foundation Trust, Canterbury, UK; 2 Department of Cardiovascular Sciences, University of Leicester, Leicester, UK; 3 Birmingham Clinical Trials Unit, School of Health Sciences, University of Birmingham, Birmingham, UK; 4 Renal Medicine, Queen Elizabeth Hospital, Birmingham, UK; 5 Institute of Inflammation and Ageing, University of Birmingham, Birmingham, UK; 6 Paediatrics, Guy's and St Thomas’ NHS Foundation Trust, London, UK; 7 Test and Prediction Research Group, Department of Applied Health Sciences, University of Birmingham, Birmingham, UK; 8 National Institute for Health and Care Research (NIHR), Birmingham Biomedical Research Centre, University of Birmingham and University Hospitals Birmingham NHS Foundation Trust, Birmingham, UK; 9 Kent Kidney Care Centre, East Kent Hospitals University NHS Foundation Trust, Canterbury, UK; 10 Department of Renal Medicine, Salford Royal Hospital, Northern Care Alliance NHS Foundation Trust, Salford, UK; 11 Diabetes Research Centre, University of Leicester, Leicester, UK; 12 Kidney Care UK, Alton, UK; 13 Centre for Health Services Studies, University of Kent, Canterbury, UK; 14 School of Medicine, University of Nottingham, Nottingham, UK; 15 Academic Unit of Health Economics, Leeds Institute of Health Sciences, University of Leeds, Leeds, UK; 16 Warwick Medical School, University of Warwick, Coventry, UK; 17 Renal Medicine, University Hospitals of Derby and Burton NHS Foundation Trust, Derby, UK

**Keywords:** Kidney failure, chronic, Nephrology, Primary health care, Biochemistry, Diagnostic tests, routine, Pathology

## Abstract

**Objective:**

To study the performance of two contemporary sets of estimating equations for glomerular filtration rate, published by CKD-EPI (Chronic Kidney Disease Epidemiology Collaboration) and EKFC (European Kidney Function Consortium) that include one (creatinine or cystatin C only) and combined (creatinine and cystatin C) biomarkers, to assess their accuracy in a population with moderate chronic kidney disease.

**Design:**

Prospective cohort study.

**Setting:**

Primary, secondary, and tertiary care in six centres in England. Participants were recruited from April 2014 to January 2017.

**Participants:**

1167 adults, aged ≥18 years, with moderate chronic kidney disease (estimated glomerular filtration rate 30-59 mL/min/1.73 m^2^ sustained over at least three months before recruitment).

**Main outcome measures:**

Accuracy of estimating equations CKD-EPI_creatinine_, CKD-EPI_cystatin_, CKD-EPI_creatinine-cystatin_, EKFC_creatinine_, EKFC_cystatin_, and EKFC_creatinine-cystatin_ compared with measured glomerular filtration rate (iohexol clearance). Remodelled 2021 versions of the CKD-EPI equations were also studied. Accuracy was expressed as P30 (percentage of estimates within 30% of measured glomerular filtration rate).

**Results:**

Median age was 67.5 years, 58.3% of patients were men, 86.9% were white participants, and 27.8% had diabetes. Median measured glomerular filtration rate was 47.0 mL/min/1.73 m^2^; 57.0% of participants had albuminuria. Test calibration critically affected measurement of cystatin C. After recalibration of cystatin C, P30 values were 90.2% (CKD-EPI_creatinine_), 89.5% (CKD-EPI_cystatin_), 94.9% (CKD-EPI_creatinine-cystatin_), 88.0% (CKD-EPI(2021)_creatinine_), 94.9% (CKD-EPI(2021)_creatinine-cystatin_), 89.4% (EKFC_creatinine_), 91.0% (EKFC_cystatin_), and 94.9% (EKFC_creatinine-cystatin_). Creatinine based equations showed varying bias depending on the glomerular filtration rate level; inclusion of cystatin C in the equations improved this effect. Differences in accuracy in age, sex, and glomerular filtration rate level subgroups varied by equation. Equations combining creatinine and cystatin performed equally across age, sex, diabetes status, albuminuria status, and body mass index categories.

**Conclusions:**

The CKD-EPI_creatinine_ equation had acceptable accuracy in a white population in England with moderate chronic kidney disease. Combined dual biomarker equations showed higher accuracy than the CKD-EPI_creatinine_ equation and their equivalent creatinine only equations. Further research is needed to determine the most accurate equation to use in people of black and South Asian origin living in England.

**WHAT IS ALREADY KNOWN ON THIS TOPIC**

- Chronic kidney disease is commonly detected and managed based on estimates of glomerular filtration rate from measurements of blood concentration of creatinine
- Creatinine based estimates of glomerular filtration rate have many limitations
- An alternative marker, cystatin C, may improve the accuracy of estimates of glomerular filtration rate

**WHAT THIS STUDY ADDS**

- The originally described (2009) and widely used CKD-EPI_creatinine_ equation (published by Chronic Kidney Disease Epidemiology Collaboration) had acceptable accuracy in a white population with moderate chronic kidney disease in England
- Provided that problems of assay standardisation can be dealt with, equations that incorporated both creatinine and cystatin C showed improved accuracy compared with single biomarker equations
- Combined biomarker equations reduced negative bias at higher levels of glomerular filtration rate and performed equally well in age, sex, albuminuria, and body mass index categories

**HOW THIS STUDY MIGHT AFFECT RESEARCH, PRACTICE, OR POLICY**

- Further research is needed to assess the relative accuracy of glomerular filtration rate equations in people of black and South Asian origin and in other minority populations in England
- Future clinical guidelines should consider the value of the improved accuracy of combined equations on clinical decision making, including eligibility for treatments (eg, sodium-glucose co-transporter 2 inhibitors) and accuracy of prescriptions (eg, chemotherapeutic agents)
- Efforts are needed to maintain the consistency of biomarker measurement over time given their increasing importance in identifying and monitoring disease

## Introduction

 Chronic kidney disease is common, with a global prevalence estimated at 9.1%.^1^ Most people with chronic kidney disease and a low excretory function have moderate (stage 3) chronic kidney disease (glomerular filtration rate 30-59 mL/min/1.73 m^2^),^2^,^4^ with an estimated prevalence in the non-institutionalised adult population in the US of 6.3%.^5^ Earlier recognition of chronic kidney disease and improved identification of those at risk of adverse outcomes enables timely intervention, leading to improved outcomes and reduced healthcare costs.^6^,^16^

Measuring glomerular filtration rate is central to the diagnosis, staging, and management of chronic kidney disease. Reference procedures for measuring glomerular filtration rate rely on clearance of an infused exogenous substance (eg, inulin, ^125^I-iothalamate, or iohexol) and are impractical for routine clinical use.^17^ Therefore, equations have been developed to estimate glomerular filtration rate based on serum creatinine concentration, with adjustments for age, sex and, in some cases, black ethnic group. The Chronic Kidney Disease Epidemiology Collaboration (CKD-EPI_creatinine_) equation is currently widely recommended for clinical use.^5 18 19^

Creatinine has many limitations as a marker of kidney function, including its relation to muscle mass and age. Measuring creatinine is also susceptible to analytical, drug, and dietary interferences. Cystatin C, a small molecular weight protein, is an alternative marker of glomerular filtration rate that is less susceptible to these problems. Cystatin C containing equations provide more accurate estimates of glomerular filtration rate than creatinine only equations in some settings.^18 20^ The National Institute for Health and Care Excellence (NICE) in England does not currently recommend cystatin C based glomerular filtration rate equations, citing insufficient high quality evidence supporting its use.^19^ In contrast, US guidelines support cystatin C use, particularly to confirm creatinine based estimated glomerular filtration rate in adults.^21^ Large prospective studies evaluating the accuracy of cystatin C in estimating glomerular filtration rate in populations with chronic kidney disease are lacking. Given the higher costs of cystatin C compared with creatinine (about £3.80 (€4.33; US$5.09) for each test compared with £0.43 for creatinine), the potential scale of testing (eg, in east Kent, UK, about 1.3 creatinine tests are undertaken annually for every member of the population; E J Lamb, personal communication, 2025), and the increasing availability of cystatin C assays on large automated laboratory test platforms, carefully validating its accuracy ahead of widespread introduction into healthcare is reasonable.

In this study, we assessed the accuracy of two sets of estimating equations for glomerular filtration rate, those published by CKD-EPI and the European Kidney Function Consortium (EKFC), in a study population recruited from six centres in England. These equations, which were mainly developed and validated in large North American and European populations, respectively, include one biomarker (creatinine or cystatin C only) equations and combined biomarker equations of both analytes.

## Methods

We conducted a prospective cohort study to compare the performance of published estimates of glomerular filtration rate with reference measurements of glomerular filtration rate.^22^ The study included adults (n=1229) with stage 3 chronic kidney disease (estimated glomerular filtration rate 30-59 mL/min/1.73 m^2^ inclusive, sustained over at least three months before recruitment) from six centres in England. The international chronic kidney disease staging system requires knowledge of both glomerular filtration rate and albuminuria to define stage. In this report, stage 3 chronic kidney disease refers to all individuals with a glomerular filtration rate of 30-59 mL/min/1.73 m^2^, irrespective of albuminuria status.

Participants attended hospital in the morning having been advised to consume a light breakfast (no meat or fish). Clinical and drug data, and information on ethnic group were recorded. Blood was taken for baseline measurements of serum creatinine and cystatin C, and a urine sample for measuring the albumin to creatinine ratio. Glomerular filtration rate was measured with an iohexol clearance method.^23^ Serum creatinine was measured by enzymatic assay and cystatin C by turbidimetric immunoassay (both on an Abbott Architect analyser, Abbott Diagnostics, https://www.abbott.co.uk/, accessed 16 October 2025) in a central laboratory. Creatinine and/or cystatin C concentrations measured on the baseline iohexol procedure sample were used to estimate glomerular filtration rate. Measured glomerular filtration rate was accepted as the reference measure against which the estimating equations for glomerular filtration rate were compared. Glomerular filtration rate was estimated with the equations CKD-EPI (CKD-EPI_creatinine_, CKD-EPI_cystatin_, and CKD-EPI_creatinine-cystatin_) and EKFC (EKFC_creatinine_, EKFC_cystatin_, and EKFC_creatinine-cystatin_).^5 20 24 25^ In 2021, CKD-EPI published revisions to the CKD-EPI equations (CKD-EPI(2021)_creatinine_ and CKD-EPI(2021)_creatinine-cystatin_), which were developed with modelling that did not include black ethnic group as a variable.^26^ For completeness, we have also included data describing the performance of these equations. The performance of some less widely used glomerular filtration rate equations in this cohort has been reported separately.^27^

During the study, we became aware of major published concerns about the positive bias of the cystatin C assay from Abbott Diagnostics. This concern was supported by an analytical recovery study (online supplementary methods). Consequently, we also measured cystatin C in a representative subset of samples (n=106) by a particle enhanced nephelometric immunoassay (Siemens BN Prospec analyser, www.siemens.com, accessed 16 October 2025) to generate adjusted cystatin C values for all samples.

Measured glomerular filtration rate was accepted as the reference measure against which estimating equations for glomerular filtration rate were compared. The performance of the glomerular filtration rate estimating equations was evaluated according to the guidelines of the National Kidney Foundation by assessing bias, precision, and accuracy of the estimated equations.^28^ Mean and median differences between estimated and measured glomerular filtration rate were calculated to provide measures of bias. Corresponding 95% confidence intervals (CIs) were calculated for mean biases. Precision was assessed by standard deviations and interquartile ranges of the differences between measured and estimated glomerular filtration rate. Accuracy was assessed by establishing the proportion of estimates of glomerular filtration rate within 30% (P30) of the iohexol measured glomerular filtration rate with corresponding 95% CIs, and also by calculating the root mean square error. R^2^ values were derived to provide a measure of agreement. Bias, precision, and accuracy were calculated for the estimated glomerular filtration rates generated with both the Abbott Diagnostics and Siemens calibrations of the cystatin C assay, and the effect of cystatin C calibration on the performance of the equations was reported.

Bias values were plotted against measured glomerular filtration rate values for each participant and a locally weighted scatterplot smoother (lowess) was fitted to the data. A line was added to the plot to indicate mean bias. The Kidney Disease: Improving Global Outcomes (KDIGO) organisation originally recommended that the CKD-EPI equations should be used, with alternative estimating equations being acceptable if shown to improve the accuracy of estimates of glomerular filtration rate.^18^ Accordingly, we compared the P30 values of the glomerular filtration rate estimating equations against the CKD-EPI equations with McNemar's test for paired data.

Exploratory analyses of the accuracy of estimated glomerular filtration rates were also undertaken by age (<50, 50-59, 60-69, 70-79, and >80 years), sex (men and women), diabetes status (diabetes or no diabetes, as recorded in the medical history), albuminuria (<3.0, 3.0-30.0, or >30.0 mg/mmol, corresponding to normal, moderately, and severely increased albuminuria in the international classification of chronic kidney disease),^29^ body mass index (<30 or ≥30, corresponding to healthy or overweight and obese or severely obese),^30^ and level of glomerular filtration rate (measured glomerular filtration rate <45 or ≥45 mL/min/1.73 m^2^, the threshold differentiating between chronic kidney disease stages 3a and 3b, respectively). Patient's data for sex were from assigned sex rather than self-reported gender. Accuracy was also studied by self-reported ethnic group (white, South Asian, or black). For the CKD-EPI_creatinine_ and CKD-EPI_creatinine-cystatin_ equations, we calculated the P30 values in black individuals with and without the adjustment factor for ethnic group, and the McNemar test was used to compare these equations.

Our target recruitment was 1300 participants, which, allowing for a dropout rate of 15-20%, would provide >90% power. We used simulation to evaluate the power of the study to detect a difference in P30 of 5%. Although the sample size focused on a primary comparison, our analysis looked at the comparison between many equations. We did not formally adjust for multiple comparisons because the estimated equations were not independent. Stata version 18.0 was used for all analyses. The online supplementary material has more details on recruitment, methods, and sample size calculation.

### Patient and public involvement

A patient, representing Kidney Care UK (www.kidneycareuk.org), was a member of the full study project management group and a further patient representative was a member of the study steering committee, which met about every six months. Both individuals provided expert patient input, recommendations on patient involvement and patient representation on study newsletters. Retention in the study was encouraged through newsletters and sending final appointment reminder letters. Participant information leaflets were prepared in collaboration with the patient representatives and were circulated for comment to patient groups at the recruiting units and to the Research Design Service southeast public patient involvement group. Recruitment and retention strategies were adjusted to meet the needs of the specific ethnic minority groups, including the production of translated material and use of translators where required for non-English speakers.

## Results

We recruited 1229 participants, and 1167 had both estimated and measured glomerular filtration rates recorded (online supplemental figure 1). Median age of participants was 67.5 years, 680 (58.3%) were men, and 1014 (86.9%) were white participants. Diabetes was a pre-existing diagnosis in 27.8% (n=324) of participants. Median measured glomerular filtration rate was 47.0 mL/min/1.73 m^2^ and 57.0% (n=665) of participants had albuminuria (albumin to creatinine ratio ≥3 mg/mmol). We found that 70 people had a measured glomerular filtration rate of <30 mL/min/1.73 m^2^ and 211 people had a measured rate ≥60 mL/min/1.73 m^2^, with a range of values from 11.9 to 103.7 mL/min/1.73 m^2^. Table 1 shows the characteristics of the study population.

**Table 1:** Characteristics of the study population

Characteristics	All patients (n=1167)*
Median (IQR) age (years)	67.5 (58.3-74.5)
No of men:women	680:487
Ethnic group:	
White	1014 (86.9)
Black	60 (5.1)
South Asian	66 (5.7)
Other†	27 (2.3)
Median (IQR) height (cm)	170 (162-176)
Median (IQR) weight (kg)	84.1 (72.5-97.3)
Median (IQR) Du Bois body surface area (m^2^)	1.96 (1.80-2.10)
Median (IQR) body mass index	29.0 (25.8-33.3)
Drug treatment recorded:	
Thiazide diuretic	123 (10.5)
Loop diuretic	180 (15.4)
Potassium sparing diuretic	26 (2.2)
β blocker	314 (26.9)
Calcium channel blocker	376 (30.6)
Angiotensin converting enzyme inhibitor	411 (35.2)
Angiotensin II receptor blocker	348 (29.8)
α blocker	153 (13.1)
Hydroxymethyl glutaryl CoA reductase inhibitor	635 (54.4)
Allopurinol	137 (11.7)
Antiplatelet drugs	367 (31.4)
Comorbidity recorded:‡	
Diabetes mellitus	324 (27.8)
Ischaemic heart disease	177 (15.2)
Angina	88 (7.5)
Heart failure	55 (4.7)
Cerebrovascular disease	85 (7.3)
Transient ischaemic attack	48 (4.1)
Stroke	37 (3.2)
Hepatitis B virus	18 (1.5)
Malignancy	191 (16.4)
Smoking status:	
Non-smoker	590 (50.6)
Current smoker	101 (8.7)
Former smoker	474 (40.6)
Unknown	2 (0.2)
Urine albumin (mg/mmol) (albumin to creatinine ratio):	
<3	483 (41.4)
3-30	396 (33.9)
>30	269 (23.1)
Missing	19 (1.6)
Median (IQR) serum creatinine (µmol/L)	129 (107-154)
Median (IQR) serum cystatin C (Abbott) (mg/L)	1.71 (1.45-2.01)
Median (IQR) serum cystatin C (Siemens) (mg/L)	1.53 (1.28-1.81)
Chronic kidney disease glomerular filtration rate stage at baseline:§	
1	7 (0.6)
2	204 (17.5)
3a	452 (38.7)
3b	434 (37.2)
4	68 (5.8)
5	2 (0.2)
Median (IQR) measured glomerular filtration rate (mL/min/1.73 m^2^)	47.0 (38.7-56.4)

Values are number (percentage) unless indicated otherwise.

* Participants with measured glomerular filtration rate and any estimated glomerular filtration rate result at baseline were included.

† Includes participants with ethnic background other than white, South Asian or black, as well as three individuals where data were not recorded.

‡ Only comorbidities affecting ≥20 individuals in the baseline recruited cohort are listed.

§ Based on measured glomerular filtration rate.

The results obtained for cystatin C with the Siemens method were closely correlated (Pearson's correlation coefficient r=0.994) with, but lower than, those obtained with the Abbott assay (online supplemental figure 2). The relation between the two methods was described by the linear regression equation Siemens=−0.08+0.94(Abbott) (online supplemental table 1). We recalculated glomerular filtration rate estimates for cystatin C containing equations, with recalibrated cystatin C values based on the linear regression equation. Recalibration of the cystatin C containing equations with Siemens data reduced bias and increased P30: for example, P30 for CKD-EPI_cystatin_ changed from 72.5 (95% CI 69.8 to 75.0) to 89.5 (87.6 to 91.2) when recalibrated values were used. The rationale for this recalibration is considered further in the discussion section. Unless stated otherwise, subsequent data reported in this paper used recalibrated (ie, Siemens) cystatin C values.

Table 2 shows the bias, precision, and accuracy of the glomerular filtration rate estimating equations. Online supplemental table 2 has equivalent data for the cystatin containing equations before cystatin C recalibration. P30 value for the CKD-EPI_creatinine_ equation was 90.2%, compared with 89.4% and 88.0% for the EKFC_creatinine_ and CKD-EPI(2021)_creatinine_ equations, respectively. Several of the cystatin C containing equations (CKD-EPI_creatinine-cystatin_, EKFC_cystatin_, EKFC_creatinine-cystatin_, and CKD-EPI(2021)_creatinine-cystatin_) had P30 values >90%. Online supplemental table 3 shows the comparative accuracy of the glomerular filtration rate equations. The CKD-EPI_creatinine-cystatin_, CKD-EPI(2021)_creatinine-cystatin_, and EKFC_creatinine-cystatin_ equations showed higher accuracy than the CKD-EPI_creatinine_ and CKD-EPI_cystatin_ single biomarker equations (P<0.001).

**Table 2:** Performance of glomerular filtration rate estimating equations compared with measured glomerular filtration rate

Equation*	Median (IQR) estimated glomerular filtration rate (mL/min/1.73 m^2^)	Bias (estimated−measured glomerular filtration rate) (**mL/min/1.73** m^2^)		R_2_	Root mean square error (mL/min/1.73 m^2^)	P30 (95% CI)
		Mean difference (SD) (95% CI)	Median difference (IQR)			
CKD-EPI_creatinine_	44.8 (36.7-53.8)	−2.5 (9.1) (−3.0 to −1.9)	−2.8 (−8.2 to 3.5)	0.55	8.83	90.2 (88.4 to 91.9)
CKD-EPI_cystatin_	42.3 (33.8-53.4)	−3.4 (9.1) (−3.9 to −2.9)	−4.1 (−9.3 to 1.5)	0.67	7.58	89.5 (87.6 to 91.2)
CKD-EPI_creatinine-cystatin_	42.7 (34.6-52.4)	−3.7 (7.3) (−4.1 to −3.3)	−3.9 (-8.4 to 1.1)	0.71	7.07	94.9 (93.5 to 96.1)
CKD-EPI(2021)_creatinine_	47.4 (38.9-56.8)	0.0 (9.3) (−0.6 to 0.5)	−0.4 (−6.0 to 6.1)	0.53	8.99	88.0 (86.0 to 89.8)
CKD-EPI(2021)_creatinine-cystatin_	45.2 (36.7-55.4)	−1.1 (7.6) (−1.5 to −0.6)	−1.3 (−6.1 to 3.7)	0.71	7.06	94.9 (93.4 to 96.1)
EKFC_creatinine_	42.8 (35.3-51.2)	−4.4 (9.0) (−4.9 to −3.8)	−4.4 (−10.0 to 1.3)	0.55	8.87	89.4 (87.5 to 91.1)
EKFC_cystatin_	46.1 (37.9-56.6)	0.1 (8.7) (−0.4 to 0.6)	−0.4 (−5.5 to 5.4)	0.65	7.77	91.0 (89.2 to 92.6)
EKFC_creatinine-cystatin_	44.6 (37.3-53.4)	−2.1 (7.3) (−2.5 to −1.7)	−2.1 (−6.8 to 2.6)	0.69	7.28	94.9 (93.4 to 96.1)

Median measured glomerular filtration rate was 47.0 mL/min/1.73 m2.

Data for equations incorporating cystatin C used values after assay recalibration. Online supplemental table 2 compares these data with those obtained before assay recalibration.

* Estimating equations for glomerular filtration rate were those published by CKD-EPI and EKFC that include one (creatinine or cystatin C only) and combined (creatinine and cystatin C) biomarkers.

† Accuracy was assessed by establishing the proportion of estimates of glomerular filtration rate within 30% (P30) of the iohexol measured glomerular filtration rate.

CI, confidence interval; CKD-EPI, Chronic Kidney Disease Epidemiology Collaboration; EKFC, European Kidney Function Consortium; IQR, interquartile range; SD, standard deviation.

**Figure 1:**
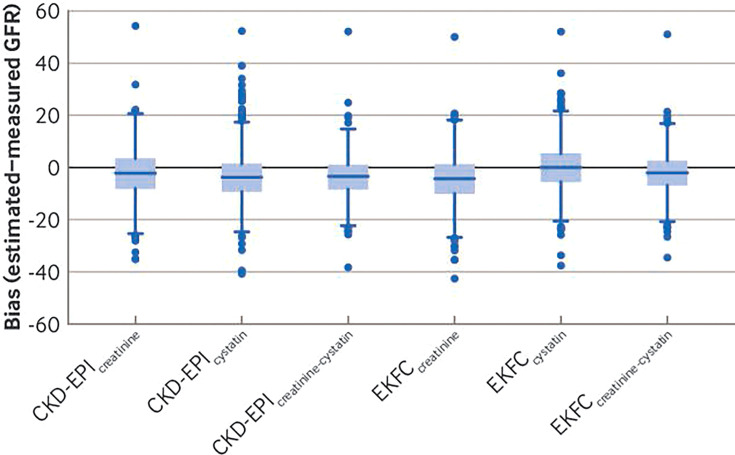
Box and whisker plots showing bias of glomerular filtration rate estimating equations compared with measured glomerular filtration rate (GFR mL/min/1.73 m^2^). Box shows median and first and third quartiles; whiskers span all data points within 1.5 interquartile range (IQR) of the nearer quartile, with Tukey outliers outside of this range (<quartile 1-1.5 IQR or >quartile 3+1.5 IQR). Estimating equations for glomerular filtration rate were those published by CKD-EPI (Chronic Kidney Disease Epidemiology Collaboration) and EKFC (European Kidney Function Consortium) that include one (creatinine or cystatin C only) and combined (creatinine and cystatin C) biomarkers. Online supplemental figure 3 shows equivalent data for CKD-EPI(2021) equations

**Figure 2:**
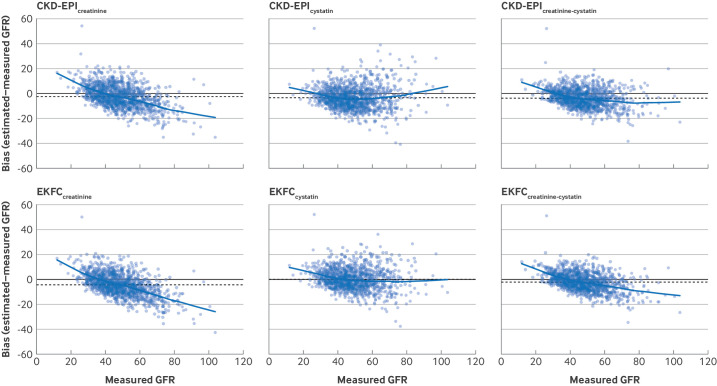
Locally weighted scatterplot smoothing (lowess) plots showing bias of glomerular filtration rate estimating equations compared with measured glomerular filtration rate (GFR mL/min/1.73 m^2^) for individual estimating equations. Estimating equations for glomerular filtration rate were those published by CKD-EPI (Chronic Kidney Disease Epidemiology Collaboration) and EKFC (European Kidney Function Consortium) that include one (creatinine or cystatin C only) and combined (creatinine and cystatin C) biomarkers. Bias plots are shown with lowess function (blue curvilinear line). Horizontal black line shows zero bias and dashed line shows mean bias. Online supplemental figure 4 shows equivalent data for CKD-EPI(2021) equations

In general, overall mean and median estimates of glomerular filtration rate showed negative bias compared with measured glomerular filtration rate (figure 1, table 2, and online supplemental figure 3). R^2^ values ranged from 0.53 to 0.71, showing moderate positive linear association. Root mean square error values ranged from 7.06 to 8.99, indicating the scale of potential error with which estimating equations approximate measured glomerular filtration rate. Use of cystatin C, particularly in the combined equations, tended to improve precision and, for the EKFC equations, reduced bias compared with measured glomerular filtration rate (figure 1 and online supplemental figure 3). Bias, however, was not constant across the range of glomerular filtration rates included in the study population. Generally, creatinine based equations showed positive bias at lower glomerular filtration rate levels (about <30-40 mL/min/1.73 m^2^) and negative bias at higher glomerular filtration rates (about >40 mL/min/1.73 m^2^), with the magnitude of the negative bias increasing as the level of glomerular filtration rate increased; this effect was partially reduced when cystatin C was incorporated into the equations (figure 2 and online supplemental figure 4).

Online supplemental table 4 presents data on the accuracy of the glomerular filtration rate estimating equations by category: age, sex, diabetes status, albuminuria status, body mass index, and glomerular filtration rate (<45 and ≥45 mL/min/1.73 m^2^). We found evidence indicating differences in accuracy in relation to some characteristics, as indicated by non-overlapping 95% CIs. The EKFC_cystatin_ equation was less accurate in those aged <50 years than in some older age groups and less accurate in women, whereas the EKFC_creatinine_ equation was less accurate in men. The accuracy of the combined biomarker equations was unaffected by sex in all cases. Diabetes, level of albuminuria, and body mass index did not affect the accuracy of any of the equations. The EKFC_cystatin_, EKFC_creatinine-cystatin_, and CKD-EPI(2021)_creatinine_ equations were less accurate in people with measured glomerular filtration rate of <45 mL/min/1.73 m^2^ than those with measured glomerular filtration rate ≥45 mL/min/1.73 m^2^.

Online supplemental table 5 shows the accuracy of the equations in white, South Asian, and black participants. We found no evidence indicating a difference in accuracy of any of the cystatin containing equations for any ethnic group, but the EKFC_creatinine_ and CKD-EPI(2021)_creatinine_ equations were less accurate in black than in white individuals, as indicated by non-overlapping 95% CIs. Removal of the black ethnic group factors from the CKD-EPI_creatinine_ and CKD-EPI_creatinine-cystatin_ equations did not significantly reduce accuracy in black people.

## Discussion

### Principal findings

We conducted a large prospective study examining how contemporary glomerular filtration rate estimating equations perform in a population recruited in England with moderate chronic kidney disease. Several equations, in particular the combined creatinine and cystatin biomarker equations, achieved clinically acceptable performance, as defined by P30 values >90%. Compared with the reference measured glomerular filtration rate, most glomerular filtration rate estimates showed negative bias overall, underestimating glomerular filtration rate, and potentially leading to over-diagnosis of chronic kidney disease stage 3. Creatinine based equations showed a particularly clear shift in bias depending on the glomerular filtration rate, tending to overestimate glomerular filtration rate at lower levels of kidney function (about 30-40 mL/min/1.73 m^2^) and underestimating glomerular filtration rate at higher levels (about >40 mL/min/1.73 m^2^). Inclusion of cystatin C in single or combined equations reduced overall bias in the EKFC equations, but not the CKD-EPI equations, and reduced, but did not eliminate, the effect of variable bias across different GFR levels of GFR. We explored potential causes for the varying performance of the equations, especially for problems related to the calibration of cystatin C.

### Comparison with other studies

The National Kidney Foundation proposed a minimal performance target for P30 of 90% for glomerular filtration rate equations, a position that was later adopted by KDIGO.^18 28^ Although many equations, particularly creatinine based equations, have struggled to achieve this benchmark,^5 24 31^ in our study the CKD-EPI_creatinine_ and EKFC_cystatin_ equations achieved P30 values >90%, similar to all combined creatinine-cystatin C equations. Combined dual biomarker equations showed higher accuracy than the CKD-EPI_creatinine_ equation and their equivalent creatinine only equations. These findings support the recent recommendations by KDIGO to use combined creatinine-cystatin C estimated glomerular filtration rate, where cystatin C testing is available, to help disease staging and in clinical situations where glomerular filtration rate based only on creatinine is known to be inaccurate.^29^

The accuracy of some of the glomerular filtration rate equations seemed to vary for some of the characteristics studied. Others have described differences in the performance of glomerular filtration rate estimating equations for varying characteristics (eg, age,^32 33^ sex,^32^ level of glomerular filtration rate,^5 32 34 35^ and body mass index).^32 33 36^ Although differences in accuracy were indicated, our study was not powered to detect differences in accuracy in the subgroups. Furthermore, our analysis did not allow for confounding factors. For equations combining creatinine and cystatin C, accuracy was the same across age, sex, diabetes status, albuminuria status, and body mass index categories.

Even when achieving P30 values >90%, the accuracy of estimated glomerular filtration rates has been criticised as being too broad for clinical decision making.^19^ How much further progress can be made in improving on this situation is uncertain. Numerous non-glomerular factors affect serum creatinine (eg, tubular secretion, extrarenal elimination, differences in skeletal muscle mass,^37^ dietary intake,^38 39^ and genetically determined differences in creatinine production rate^40^) and cystatin C (eg, lean mass,^41^ glucocorticoid treatment,^42^ smoking status,^43 44^ and genetic influences^45^) concentrations that estimating equations cannot account for. Improved creatinine standardisation and method of adjusting glomerular filtration rate for body surface area could achieve minor improvements in the accuracy of glomerular filtration rate estimates.^27^ A problem that will affect the upper limits of accuracy of any approach, however, is the biological and analytical variability of the reference measurement procedure itself; both estimated and measured glomerular filtration rates have error compared with the true glomerular filtration rate.^23^

A major source of inaccuracy in our study was the substantial positive bias of the Abbott Diagnostics cystatin C method, resulting in major negative bias of cystatin containing glomerular filtration rate estimating equations (online supplemental table 2). Although cystatin C assays are calibrated against an international reference preparation (ERM-DA471/IFCC),^46^ evidence indicated discordance between different manufacturers' methods, including a substantial positive bias of 16-20% in the Abbott Diagnostics cystatin C assay.^47 48^ Our data confirmed over-recovery of 12.4% in the Abbott Diagnostics cystatin C assay, sufficient to cause the observed negative bias in the glomerular filtration rate estimates. Our comparison study with the Siemens cystatin C assay further supported this finding. After careful consideration, we recalibrated our Abbott Diagnostics cystatin C data against the Siemens method to ensure that our study data represented the performance of cystatin C based glomerular filtration rate estimating equations under internationally standardised conditions. This recalibration substantially improved the negative bias of the cystatin C containing estimating equations, showing superior accuracy of combined biomarker equations compared with single biomarker creatinine containing equations. Our observation of over-recovery by the Abbott Diagnostics assay highlights the crucial importance of standardisation of cystatin C assays for both clinical and research applications. Online supplementary material gives more details on the Abbott Diagnostics calibration issue.

The performance of glomerular filtration rate estimating equations in ethnic minority groups has always been an area of debate. Concerns exist that adjustment for black race in the CKD-EPI equations may have contributed to falsely high glomerular filtration rate estimations among black people.^19 21^ Potentially, adjustment for black race may have exacerbated pre-existing inequalities in access to healthcare in some individuals (eg, access to advanced kidney care planning and some drugs that are prescribed based on glomerular filtration rate level).^26^ Recently, the National Kidney Foundation-American Society of Nephrology recommended that black race adjustment factors should no longer be used and revised CKD-EPI(2021) equations were published that had been remodelled without this factor.^21 26^ Similarly, the 2021 NICE guideline on chronic kidney disease removed the recommendation to adjust for black ethnic group and called for further research to establish which glomerular filtration rate estimations are the most accurate in people from black, Asian, and other minority ethnic groups living in England with chronic kidney disease.^19^ Our study cannot adequately answer this question. Recruitment was challenging among South Asian and black populations. Although no consistent reasons emerged to explain why patients from these ethnic groups declined participation, lower rates were in keeping with previous studies.^49 50^ The overall percentage of non-white participants in our study was below that recorded from recent English census data (13.1% compared with 19.0%),^51^ and our data were not powered to detect differences between ethnic groups. Nevertheless, we found provisional evidence indicating reduced accuracy of the EKFC_creatinine_ and CKD-EPI(2021)_creatinine_ equations among black compared with white people (online supplemental table 4). Further research will be required to fully look at this question.

### Strengths and limitations of this study

The strengths of our study included a large cohort and the use of a reference glomerular filtration rate test that included a three point iohexol clearance procedure, with the final sample taken four hours after injection. Although this time interval has generally been considered suitable for patients with glomerular filtration rates of >30 mL/min/1.73 m^2^,^52^ recent evidence suggests that this threshold is too low and that patients with higher glomerular filtration rates should also be tested with an extended clearance period.^53^ Not using an extended collection period in individuals with a low glomerular filtration rate could lead to overestimation of the glomerular filtration rate. Although we acknowledge the recent efforts of EKFC to standardise iohexol clearance procedures,^53^ our use of three time points enabled confirmation of a linear reduction in iohexol concentration with regression analysis (r>0.99 in most study participants), with no evidence indicating that delayed clearance in those with a lower glomerular filtration rate was a problem. A major reason given by patients for declining to participate in the study was the length of the measured glomerular filtration rate appointment; we believe that prolonging the test duration, from 240 to 420 min, as recommended,^53^ would have adversely affected recruitment to the study.

All analytical methods used in the study were rigorously quality assured. Creatinine and cystatin C were measured in centralised laboratories to eliminate variability between laboratories. We used an enzymatic creatinine method, which is less prone to interference than the widely used Jaffe methods, and its specificity facilitates standardisation against isotope dilution-mass spectrometry reference methodology. Participants were asked to avoid eating meat and fish on test days, because these foods can acutely increase blood levels of creatinine, thus suppressing the estimated glomerular filtration rate.^38^ Although these stringent analytical and patient preparation procedures improved data accuracy, these idealised sampling conditions may not reflect the clinical situation. We also explored the problem of cystatin C calibration on the performance of the glomerular filtration rate equation to an extent not usually considered in such studies. Within the context of the research study, we could study and adjust accordingly the calibration of our commercial cystatin C method, an adjustment that is not available to most clinical diagnostic laboratories.

By design, our study was limited to chronic kidney disease stage 3, although at baseline, 26% of those recruited had a measured glomerular filtration rate outside of the range 30-59 mL/min/1.73 m^2^. This variance is expected given the biological variation and performance characteristics of glomerular filtration rate estimating equations compared with measured glomerular filtration rate. For example, if a glomerular filtration rate estimating equation has a P30 of 90%, for individuals with a measured glomerular filtration rate of 59 mL/min/1.73 m^2^, the estimated glomerular filtration rate will lie within 41 and 77 mL/min/1.73 m^2^ 90% of the time; in 10% of individuals the rate will fall outside of this range. The inclusion of individuals with glomerular filtration rates outside the 30-59 mL/min/1.73 m^2^ range allowed for broader observations to be made about the performance of the equations outside of stage 3 chronic kidney disease, but the strength of any conclusions in this respect are necessarily limited.

### Areas of further research

Our findings showed that incorporation of cystatin C into creatinine containing equations improved the accuracy of the estimates of glomerular filtration rate in patients with moderate chronic kidney disease and reduced bias at higher levels of glomerular filtration rate. Extending this observation by studying patients with a mildly reduced glomerular filtration rate (60-89 mL/min/1.73 m^2^) would be useful. Inclusion of cystatin C in the glomerular filtration rate estimation may offer advantages over those based only on creatinine in other groups, in particular because of the relation of creatinine with muscle mass. These populations would include children and people with unusual muscle mass (eg, people with amputated limbs, people with advanced malignancy, athletes, and body builders). A growing concern is how best to estimate glomerular filtration rate in transgender people, where the assignment of sex can complicate estimation of glomerular filtration rate. Combined biomarker equations did not seem to be influenced by sex and may therefore offer advantages in this scenario. Further research in these populations is warranted. Assessment of glomerular filtration rate in ethnic minority groups remains a crucial question. The relative independence of cystatin C concentration from racial influence seems to offer advantages in this respect.

The use of cystatin C modestly increases the economic cost of managing chronic kidney disease. Implementation requires proof of cost effectiveness, but little evidence exists.^27^ For example, recent guidelines on the use of hypoglycaemic drug treatments (sodium-glucose co-transporter 2 inhibitors) in adults with chronic kidney disease and type 2 diabetes are dependent on albuminuria and estimated glomerular filtration rate thresholds.^19^ Research in this context may help quantify the effect of more accurate assessment of glomerular filtration rate on longer term costs and outcomes. A theoretical modelling study from Northern Europe has recently confirmed improved decision making in relation to eligibility for sodium-glucose co-transporter 2 inhibitors (in addition to improved accuracy of doses of several other medicines) when combined biomarker equations were used compared with those based on creatinine only.^36^

### Conclusions

Our observations in an English population with moderate chronic kidney disease confirmed that all glomerular filtration rate estimating equations showed variable bias compared with measured glomerular filtration rate. If only serum creatinine is available, the CKD-EPI equation had acceptable accuracy in a white population. Although questions of assay standardisation remain, if serum cystatin C is also available, equations combining creatinine and cystatin C can offer improved accuracy and perform more consistently in terms of bias for a wider range of glomerular filtration rate levels. Further research is needed to determine the most accurate equation to use in people of black and South Asian origin in England.

## Supplementary material

10.1136/bmjmed-2025-001827 Supplementary Figure 1

10.1136/bmjmed-2025-001827 Supplementary Figure 2

10.1136/bmjmed-2025-001827 Supplementary Figure 3

10.1136/bmjmed-2025-001827 Supplementary Figure 4

10.1136/bmjmed-2025-001827 Supplementary file 1

## Data Availability

Data are available in a public, open access repository.
