# Supplementary material for: Test accuracy of glomerular filtration rate estimation with creatinine and cystatin C in adults with moderate chronic kidney disease: prospective cohort study
Source: BMJ Med. 2026 Jan 21;5(1):e001827. doi: 10.1136/bmjmed-2025-001827 (PMC12829397; doi:10.1136/bmjmed-2025-001827)
Supplement: Supplementary Figure 1 [file bmjmed-5-1-s001.pdf]

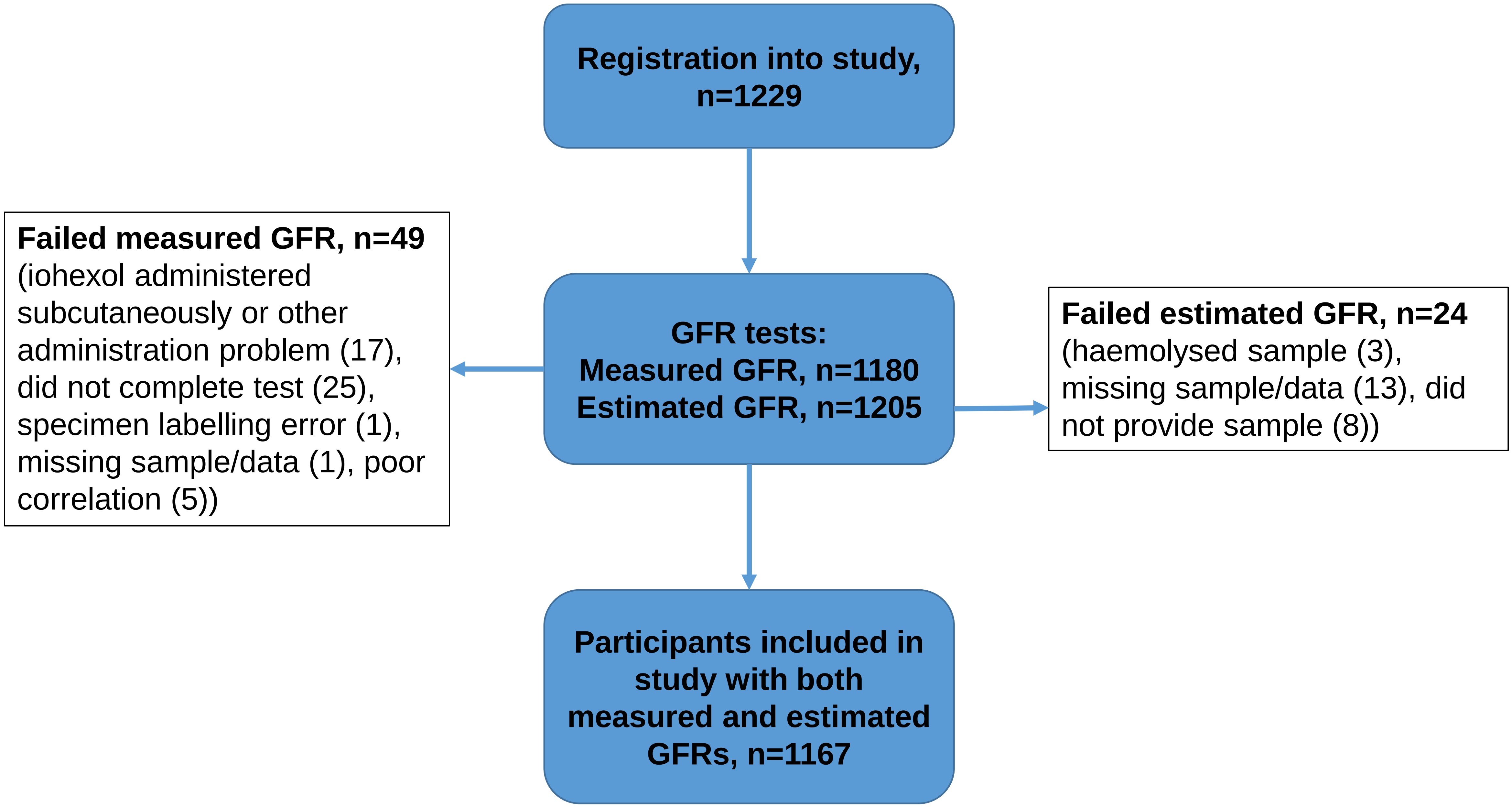
Registration into study, n=1229
GFR tests: Measured GFR, n=1180 Estimated GFR, n=1205
Failed measured GFR, n=49 (iohexol administered subcutaneously or other administration problem (17), did not complete test (25), specimen labelling error (1), missing sample/data (1), poor correlation (5))
Failed estimated GFR, n=24 (haemolysed sample (3), missing sample/data (13), did not provide sample (8))
Participants included in study with both measured and estimated GFRs, n=1167
