# Supplementary figures and images for: Test accuracy of glomerular filtration rate estimation with creatinine and cystatin C in adults with moderate chronic kidney disease: prospective cohort study
Source: BMJ Med. 2026 Jan 21;5(1):e001827. doi: 10.1136/bmjmed-2025-001827 (PMC12829397; doi:10.1136/bmjmed-2025-001827)

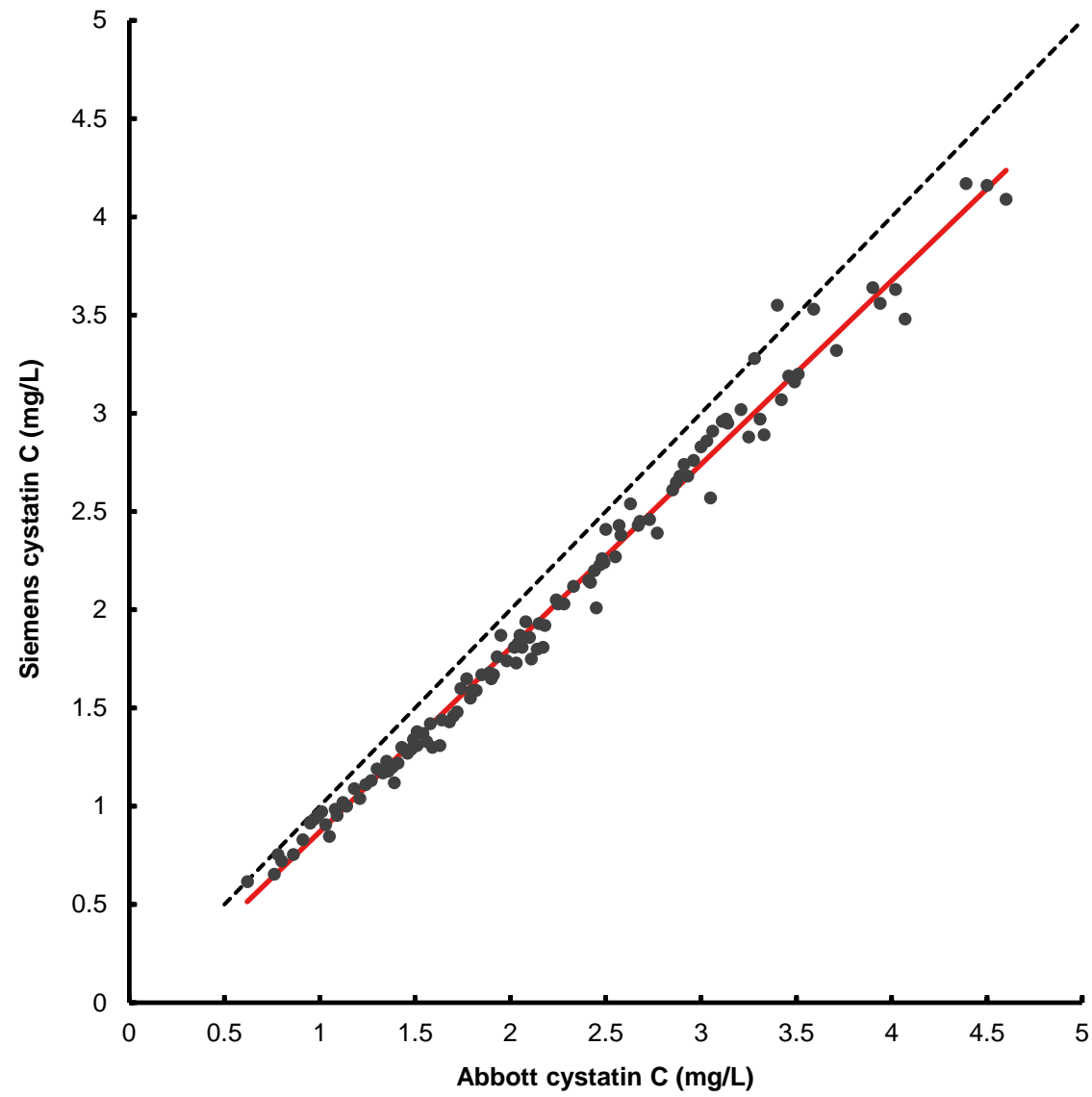

Supplement: Supplementary Figure 2 [file bmjmed-5-1-s002.pdf]

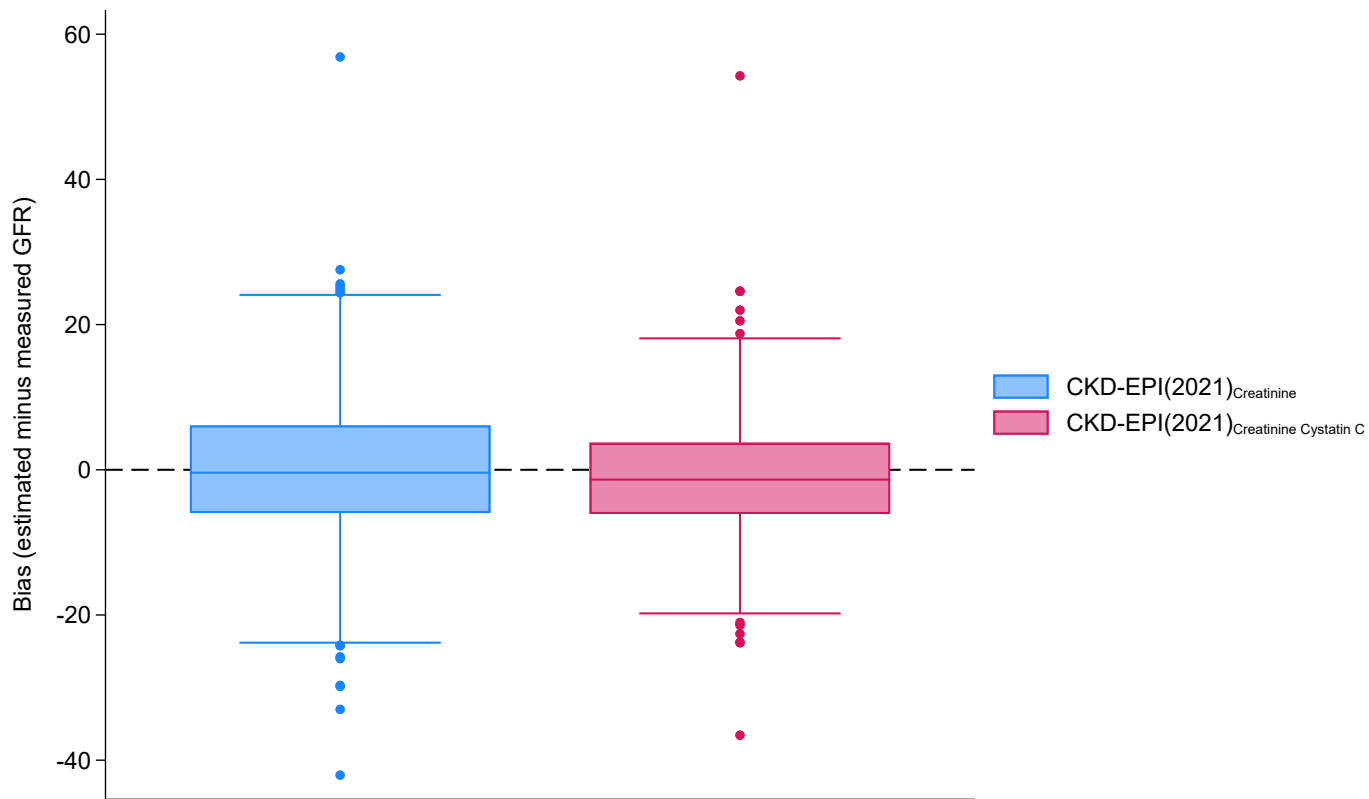

Supplement: Supplementary Figure 3 [file bmjmed-5-1-s003.pdf]

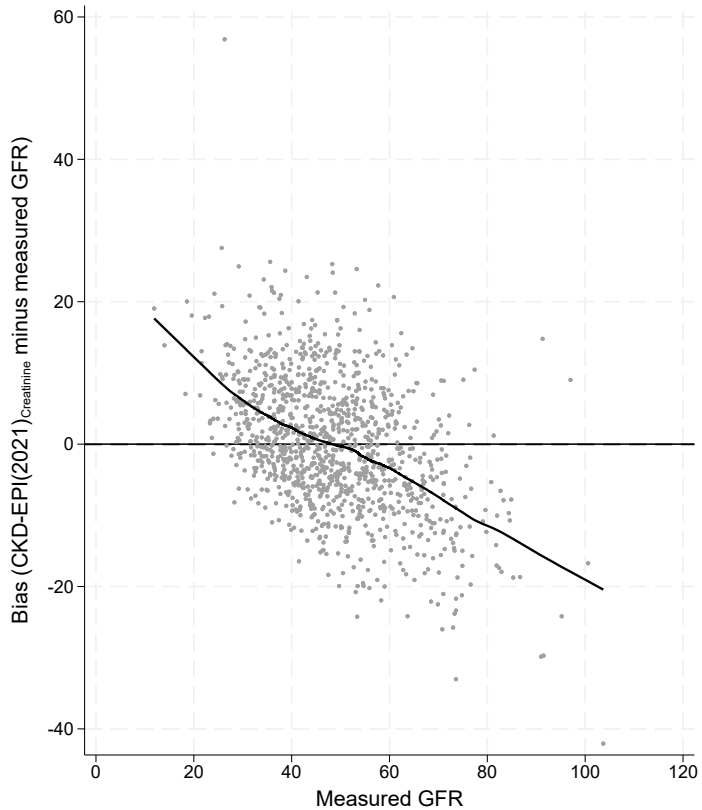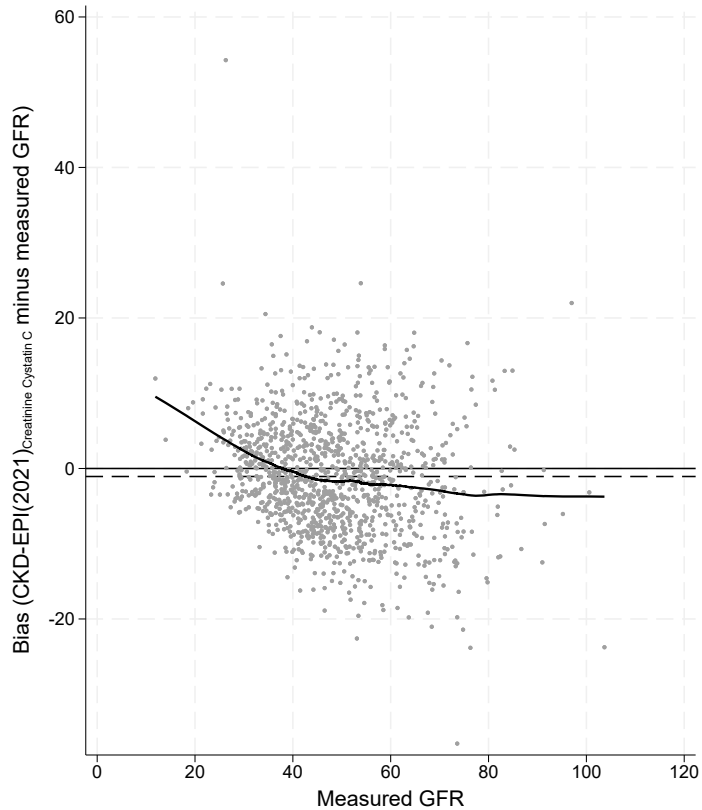

Supplement: Supplementary Figure 4 [file bmjmed-5-1-s004.pdf]
