# Supplementary material for: Test accuracy of glomerular filtration rate estimation with creatinine and cystatin C in adults with moderate chronic kidney disease: prospective cohort study
Source: BMJ Med. 2026 Jan 21;5(1):e001827. doi: 10.1136/bmjmed-2025-001827 (PMC12829397; doi:10.1136/bmjmed-2025-001827)
Supplement: Supplementary file 1 [file bmjmed-5-1-s005.pdf]

Supplementary file for Lamb EJ et al, Glomerular filtration rate estimation using creatinine and cystatin C: a prospective study of test accuracy in adults with moderate chronic kidney disease

Supplementary Methods

Sample size calculation

We evaluated the ability of the study to detect a difference between two equations of 5% in P30, between 81% and 86%, which is of a magnitude considered clinically important and likely to occur with the expected scale of differences in imprecision between the equations. With 1000 evaluable subjects our simulations showed 87% power for detecting a difference at the 5% level. We thus aimed to recruit 1300 people, which allowing for 15-20% drop-out would deliver over 90% power. This calculation was conservative in that it only took imprecision into account. In the presence of systematic bias (a reasonable assumption) then the power was estimated to be greater than this. The sample size focused on a primary comparison but our analysis addresses the comparison between many equations. We did not formally adjust for multiple comparisons as the estimated equations were not independent.

Recruitment

The aim of the study was to recruit 1300 individuals in total. The first participant was recruited in April 2014. It was anticipated that with six recruiting centres a recruitment rate of approximately 72 participants per month was realistic, but this was difficult to achieve. Recruitment of South-Asian and black individuals was also more difficult than expected, and target population sizes had to be modified accordingly. Recruitment and retention was reviewed and discussed regularly at study management group meetings. Various changes to the study were made to improve recruitment, including extending primary care recruitment beyond the two centres originally envisaged, modifying and translating participant information sheets. Retention in the study was encouraged through newsletters and sending final appointment reminder letters. In January 2017 revised sample size/power calculations indicated that under the proposed analysis method for the primary outcomes, with 1,167 evaluable subjects the study had greater than 87% power to detect a 5% difference in P30 between equations. Following discussion between the study management group, the independent study steering committee and the funder, recruitment was terminated due to low ongoing recruitment rates.

In total 29,845 people were screened for potential suitability for study inclusion: 15,340 were deemed unsuitable at an early stage from informatics and clinic lists due to not having CKD stage 3. Other identified reasons for unsuitability included that they were too unwell, had recently had acute kidney injury (AKI), were unable to consent, were known alcohol or drug abusers, had had a previous reaction to iodine, were amputees, were under 18 years of age or were pregnant or breast feeding. People considered suitable for study inclusion (n=6,209) were approached in person and/or sent participant information sheets. A further 4,000 individuals were contacted through their primary care provider. Reasons for declining to participate were recorded in 928 cases. The major reasons for declining included that they were not interested in research; that they had too many medical appointments; that the five

hour appointment time was too long; that too much travel was involved; that they were already in other research studies; and that the study involved too many injections.

A total of 1,229 participants were recruited to the study between April 2014 and January 2017. Recruitment was primarily from secondary/tertiary care, with 72 patients being recruited from primary care. Of the 1,229 participants recruited to the study, 1,205 and 1,180 respectively had evaluable estimated and measured GFRs at baseline, with 1,167 (95.0%) having both estimated and measured GFRs recorded (Supplementary Figure 1).

Adults with stage 3 CKD were recruited at six centres in England (Birmingham, Canterbury, Derby, Leicester, Salford and London [Kings College Hospital]). Participants were recruited from both primary and secondary/tertiary care. Conservative estimates of stage 3 CKD population prevalence in East Kent and Leicestershire, for example, are 4.0% and 5.9% respectively.[1, 2] Individuals aged 18 years and older having stage 3 CKD (estimated GFR measurements between 30 and 59 mL/min/1.73 m^2 inclusive, obtained using the Modification of Diet in Renal Disease (MDRD) Study equation,[3] sustained over at least 3 months prior to recruitment) were included. The MDRD equation was used as this was standard practice in England at the time of study commencement. Exclusions were a history of untoward reaction to iodinated contrast media or allergy to topical iodine, pregnant or breast-feeding, known current alcohol or drug abuse, kidney transplant recipient, people whose life expectancy would make study completion unlikely,[4] inability to consent e.g. due to cognitive impairment, inability to comply with study schedule and follow-up, amputation of whole or part limb, recent (last 6 months) episode of AKI,[5] and sickle cell disease.

Sampling and data collection

Participants were asked to attend hospital in the morning having been advised to consume a light breakfast (no meat or fish). A clinical and drug history was recorded using a standardised questionnaire taken by research nurses on the day of hospital attendance. Vascular disease was defined as the presence of myocardial infarction (MI, including ST-elevation myocardial infarction, [STEMI] and non-ST elevation myocardial infarction [NSTEMI]), angina, congestive cardiac failure (heart failure) or a requirement for coronary intervention (angioplasty, coronary artery bypass graft or pacemaker), cerebrovascular or peripheral vascular disease. Information on ethnicity was gathered using a modified version of the 2011 UK Census Questionnaire, with ethnicity being mapped to the following codes: Caucasian 31, 32, 33, 34; South-Asian 39, 40, 41; Black 44, 45, 46. Height was measured to the nearest 0.1 cm with a rigid stadiometer. Body weight was measured in light indoor clothing to the nearest 0.1 kg. Brachial blood pressure was measured as recommended by the British and Irish Hypertension Society (https://bihs.org.uk/ , accessed 16th October 2025) three times in the sitting position using standardised Omron M7 digital sphygmomanometers (Omron Healthcare, Milton Keynes, UK). The average of the second and third blood pressure readings was recorded.

Baseline blood was taken for serum creatinine and cystatin C, and a urine sample was collected for albumin to creatinine ratio (ACR). Blood samples were collected using standard venepuncture and phlebotomy procedures including the use of a tourniquet. Blood was collected in appropriate Greiner Vacuette™ tubes (www.gbo.com, accessed 16th October 2025) following the manufacturer's recommended order of draw. The urine sample was taken into a plain sterilin pot. Samples were transported to the local laboratory, where

plasma/serum was separated within 4-6 h of venepuncture by centrifuging at 2000 g for 10 mins. Aliquots of serum/plasma and urine were then stored at -80°C pending transportation to the central laboratories (St. Thomas's Hospital, London [iohexol, isotope-dilution mass spectrometry (ID-MS) creatinine] or Canterbury [enzymatic creatinine, cystatin C, albumin to creatinine ratio (ACR)] depending on analyte) and analysis.

GFR was measured using an iohexol clearance method.[6] A 5 mL bolus of Omnipaque 240 (518 g/L iohexol corresponding to 240 g/L of iodine, GE Healthcare (https://www.gehealthcare.co.uk/, accessed 16th October 2025) followed by 10 mL of normal saline was injected into the antecubital vein. Blood samples were collected at 5, 120, 180 and 240 minutes after injection. Exact time of the samples in relation to the bolus injection was accurately recorded. Participants were allowed free access to fluids during the collection procedure but were asked to refrain from protein intake (i.e. biscuits/toast would be permitted) and to refrain from excessive exercise. Samples were stored at -80°C prior to analysis. Iohexol was determined using an ID-MS method (see below) and GFR calculated.[7]

GFR was estimated using serum creatinine and/or cystatin C results measured on the baseline blood sample obtained during the iohexol procedure. GFR was estimated using the following equations: the Chronic Kidney Disease Epidemiology Collaboration ($CKD\text{-}EPI_{creatinine}$) equation,[8] the CKD-EPI cystatin C-containing equations ($CKD\text{-}EPI_{cystatin}$, $CKD\text{-}EPI_{creatinine\text{-}cystatin}$),[9] the European Kidney Function Consortium ($EKFC_{creatinine}$, $EKFC_{cystatin}$, $EKFC_{creatinine\text{-}cystatin}$) equations,[10, 11] and the 2021 revisions of the CKD-EPI equations ($CKD\text{-}EPI(2021)_{creatinine}$ and $CKD\text{-}EPI(2021)_{creatinine\text{-}cystatin}$).[12]

Laboratory analyses

Iohexol and creatinine were measured simultaneously using electrospray ID-MS on an ABSCIEX API6500 Q-trap (ABSCIEX, Warrington, UK) mass spectrometer,[6, 13] with minor modification (see below). Iohexol stock standard, 10 mmol/L, was prepared by diluting Omnipaque 300 solution (647 g/L) in deionised water and stored in 1 mL aliquots at -80°C. In addition, an aqueous 1 mmol/L iohexol standard was prepared from solid iohexol (European Pharmacopoeia Reference Standard, Merck Life Sciences UK, Gillingham, UK, Code:I0320800, Batch 2.0, Id:000I1U, 0558) and stored in 1 mL aliquots at -80°C. Aqueous stock creatinine standard (10 mmol/L) was prepared as described previously and stored in 1 mL aliquots at -80°C.[13] Mixed aqueous calibrators: iohexol, 0, 10, 100, 500 µmol/L, creatinine, 0, 25, 100, 500 µmol/L were prepared from the stock iohexol and creatinine standards by dilution and stored in 0.5 mL aliquots at -80°C. As an assay calibration check, the iohexol reference standard was diluted to 100 µmol/L and stored in 0.5 mL aliquots at -80°C. Iohexol stable isotope ($^2H_5$-iohexol, Toronto Research Chemicals, obtained from 2BScientific Ltd, Upper Heyford, UK) was dissolved in deionised water at circa 10 mmol/L, and stored at -80°C. Plasma control samples were prepared by spiking a plasma pool with iohexol stock standard at 10, 100, and 400 µmol/L and creatinine stock standard at 0, 100, 400 µmol/L and stored in 0.5 mL aliquots at -80°C. Calibrators, controls, patient samples and stable isotope stock solutions were thawed from frozen on a roller mixer at room temperature for no more than 60 minutes, and then centrifuged for 4 minutes at 1,500 g at 4°C (Eppendorf 5810R centrifuge, VWR International Ltd, Lutterworth, UK). An aqueous working iohexol/creatinine stable isotope internal standard solution was prepared by diluting the circa 10 mmol/L $^2H_5$-iohexol solution and 10 mmol/L $^2H_3$-creatinine 1:200 with deionised

water. Calibrators, controls, and samples were pipetted (10 µL) into 2 mL microcentrifuge tubes (000-MICR-200, Elkay Laboratory Products (UK) Ltd, Basingstoke, UK) and 50 µL working stable isotope, followed by 200 µL acetonitrile (Rathburn Chemicals Ltd, Walkerburn, UK) were added to each tube. Samples were capped, vortex mixed for 5 seconds and centrifuged for 5 minutes at 20,800 g at 4°C (Eppendorf 5417R centrifuge, VWR International Ltd, Lutterworth, UK). Supernatants (200 µL) were then transferred into a 96 deep well plate and loaded onto the autosampler. Sample (2 µL) was automatically injected into a mobile phase stream of acetonitrile:water (1:1) with 0.025% formic acid using a Hewlett-Packard 1100 Series autosampler and pump (Applied Biosystems, Warrington, UK) at 250 µL/minute. Chromatography was performed on a Chirobiotic T 100 x 2.1 mm column with a 2 cm x 4.0 mm guard column (Sigma-Aldrich Company Ltd, Poole, UK).

Tandem mass spectrometry was performed in positive ion multiple reaction monitoring (MRM) mode: iohexol 821.849/602.8, $^{2}H_{5}$-iohexol 826.849/607.8, creatinine 114.16/44.0, $^{2}H_{3}$-creatinine 117.16/47.0. Dwell time was 50 msec/MRM with a 5.007 msec delay between each MRM. Total data acquisition time was 6 minutes. Iohexol and creatinine concentrations were calculated in Analyst 1.6 (ABSCIEX, Warrington, UK) using the ratio of sample peak area to stable isotope peak area. Between-day imprecision (coefficient of variation, %) for iohexol was 5.4%, 3.3% and 2.8% at 10, 100 and 400 umol/L, respectively. The laboratory participated in an international proficiency testing scheme (EQUALIS, https://equalis.se/en/, accessed 16th October 2025) for iohexol measurement with good performance. Between-day imprecision (coefficient of variation, %) for creatinine was 1.5%, 1.2% and 0.8% at 60, 160 and 460 umol/L, respectively. Please note that ID-MS creatinine data are not reported in this manuscript.

Iohexol concentrations were log transformed (natural log) and plotted as a function of time. GFR was calculated from the slope-intercept method using a single compartment model,[14, 15]

GFR (mL/min) = 0.693 x iohexol volume of distribution (L) x 1000/half-life of iohexol (min)

To ensure integrity of the iohexol procedure (dose administration, sample collection, sample labelling, and iohexol analysis) the iohexol data were rigorously reviewed for every measured GFR. The 5 min sample enabled identification of procedures where the iohexol was given subcutaneously in error, or where saline flushing of the infusion line was sub-optimal, as demonstrated by low and high iohexol concentrations respectively. In addition, all procedures where the iohexol concentration versus time correlation coefficient (r) was <0.98 (<6% of total procedures) were re-analysed to check for any within-assay sample transposition. GFR was adjusted for body surface area (BSA) using the Du Bois equation[16] and corrected for the fast exponential.[7]

Serum creatinine was measured using an enzymatic assay on an Abbott Architect analyser (Abbott Diagnostics Ltd, https://www.abbott.co.uk/, accessed 16th October 2025) standardised to the reference material, NIST SRM 967 and 914. Between-day imprecision (coefficient of variation, %) was 0.8%, 0.3% and 0.4% at concentrations of 75, 176 and 760 umol/L respectively. The laboratory participated in an international proficiency testing scheme (UKNEQAS, https://birminghamquality.org.uk/, accessed 16th October 2025) for creatinine measurement and GFR estimation with satisfactory performance.

Cystatin C was measured by a turbidimetric immunoassay on an Abbott Architect analyser calibrated against the international certified reference material ERM-DA471/IFCC for cystatin C.[17] Between-day imprecision was 2.3% and 1.6% at concentrations of 0.9 and 4.0 mg/L respectively. The laboratory participated in an international proficiency testing scheme (EQUALIS, https://equalis.se/en/, accessed 16th October 2025) for cystatin C measurement and GFR estimation with good performance.

Prior to analysis, samples were thawed at room temperature, mixed by inversion and centrifuged. Each of the biomarker analyses was undertaken by a single operator blinded to participant data using a single instrument. Creatinine and cystatin C measurements were undertaken in an accredited laboratory by scientists registered with the Health and Care Professions Council and blinded to the results of the reference test.

During the course of the study we became aware of published data describing a significant positive bias of the Abbott cystatin C assay.[18, 19] This was supported by information from the EQUALIS proficiency testing scheme and our own re-analysis of historical stored samples (data not presented here). To investigate this, a recovery study was undertaken in which lyophilised human serum cystatin C ERM-DA471/IFCC (nominal concentration 5.48 mg/L, Sigma-Aldrich Chemical Co., sigmaaldrich.com, accessed 16th October 2025) was reconstituted in 1.0 mL distilled water. The nominal concentration was adjusted by taking into account the weight of the vial before and after reconstitution. Cystatin C was then diluted x2, x3, x5, x10 in pooled non-uraemic (creatinine <80 umol/L, cystatin C 0.91 mg/L) serum to give samples with a range of expected concentrations covering 1.47 to 3.41 mg/L. These samples were analysed and the mean recovery calculated compared to the same series of samples that had had an equivalent volume of distilled water added. This recovery study was originally undertaken in February 2018 when we observed average recovery of cystatin C ERM-DA471/IFCC to be 112.4%. We repeated this study in May 2024: on this occasion average recovery was 109.3%.

To further explore this bias, in a subset of samples (n=106) covering a representative range of concentrations of cystatin C was also measured by a particle-enhanced nephelometric immunoassay according to the manufacturer's instructions on a Siemens BN Prospec analyser (www.siemens.com, accessed 16th October 2025). The Siemens assay was also calibrated against ERM-DA471/IFCC for cystatin C.[17] Between batch imprecision (n=38) for the Siemens assay was 3.5% at 0.87 mg/L and 3.6% at 4.64 mg/L. These observations were analysed using both Deming regression and linear regression analyses to generate an equation to adjust the Abbott results in the entire study cohort to resemble Siemens results. These adjusted results were then used to estimate GFR in equations incorporating cystatin C which could be compared to measured GFR as above.

<u>Funding, registration, ethical approval and patient involvement</u>

The study proposal was developed in response to a commissioning brief from the National Institute for Health and Care Research (NIHR) Health Technology Assessment (HTA) programme entitled "Cystatin C test in the assessment of renal function". The NIHR commissioned the study having gone through rigorous prioritisation assessments by national funders and national health funding bodies. These prioritisation processes ensure that studies of wide health importance and impact are funded and completed. In the UK, as a major funder, the NIHR chooses important studies on the basis of NHS and patient needs

(which is also part of the process used by NICE, the National Institute for Health and Care Excellence, to make guidelines including further research requirements). NIHR commissioned this study on the basis of NICE recommendations that a study of this nature was needed to make health decisions based on accurate diagnostic testing. The outline application was submitted in February 2012 and subsequently revised in response to comments from the NIHR's commissioning board and reviewers before final approval in July 2013. The HTA award, grant reference 11/103/01, commenced on 1st August 2013. During the study, the research team submitted 6-mothly progress reports to the HTA and met with the funder on occasion, in particular to discuss recruitment issues (see above).

The study was registered as ISRCTN42955626 http://www.controlled-trials.com/ISRCTN42955626 (accessed 16th October 2025).

Ethical approval for this study was obtained from the National Research Ethics Service (NRES Committee South East Coast – Surrey, reference 13/LO/1349, approved 9th October 2013). Written informed consent was obtained from all participants.

FCL (Director of the former Kidney Alliance and subsequently Policy Director of Kidney Care UK (https://www.kidneycareuk.org/, accessed 16th October 2025) was a full member of the study group and provided lay input, recommendations on patient involvement and patient representation on participation sheets and study newsletters. Participant information leaflets were prepared in collaboration with FCL and were circulated for comment to patient groups at the recruiting units and to the Research Design Service south-east public patient involvement group. Recruitment and retention strategies were adjusted to meet the needs of the specific ethnic minority groups including the production of translated material and use of translators where required for non-English speakers.

Supplementary Results and Discussion

The impact of calibration on cystatin C and GFR estimation

Using the Abbott cystatin C assay we observed significant negative bias of CKD-EPI$_{cystatin}$ and CKD-EPI$_{creatinine\text{-}cystatin}$ GFR estimating equations. This was unexpected based on earlier data from members of the study group.[20] Cystatin C assays are calibrated against an international reference preparation (ERM-DA471/IFCC) developed with the aim of achieving improved agreement between assays from different manufacturers and ensuring consistency across time.[17] ERM-DA471/IFCC was originally verified as being commutable for use in the Abbott immunoassay.[17] However, during the course of this study evidence emerged of continuing discordance between different manufacturers methods.[18] A report in 2017 described a significant positive bias of some 16-20% of the Abbott cystatin C assay, resulting in significantly negatively biased GFR estimates.[19] Communication with Abbott Diagnostics confirmed that they had seen a shift in their calibration but that the assay remained within their manufacturing tolerance.

We further explored this issue through a laboratory recovery study, which confirmed an average over-recovery of 12.4% in the Abbott cystatin C assay. We also undertook a comparison study against the Siemens BN Prospec assay, which further supported a positive bias of the Abbott assay (Supplementary Table 1, Supplementary Figure 2). The positive bias of the cystatin C assay was sufficient to cause significant negative bias of GFR estimates (Supplementary Table 2). The Siemens method on the BN Prospec analyser is

reportedly the only commercially available assay achieving prespecified performance criteria in relation to bias and precision.[19] After careful consideration, we decided that our Abbott cystatin C data should be recalibrated against the Siemens BN Prospec assay, to ensure that our study data represented the performance of cystatin C-based GFR estimating equations under internationally standardised conditions. Following recalibration, the negative bias of the CKD-EPI and EKFC cystatin-containing equations was significantly improved and indistinguishable from that of the $\text{CKD-EPI}_{\text{creatinine}}$ equation (main paper, Table 2).

The positive bias of the Abbott cystatin C assay illustrates the difficulty when transferring reference calibrator values to field methods. The issues are particularly complex with immunoassay where the major “reagent” is antibody based. There are multiple potential sources of variation. For example there may be subtle differences in the antigens that different manufacturers use to inoculate the antibody producing animals. The animal species used to make the antibodies may also influence speed and affinity of the reaction with the antigen. Consequently, antibodies used by different manufacturers may have differing selectivity, affinity and avidity for cystatin C in the sample, may be differentially affected by matrix effects present in human serum samples (other proteins, salt, phospholipids, complement, drugs and other substances), and possibly also by genetic variation.[21] This issue is important: there is an assumption that the introduction of the international standard for cystatin C has resulted in globally aligned assays. Although there is evidence that between method agreement has improved following introduction of the standard,[22] evidence from this study and others indicates that further efforts are required to improve assay comparability in this area.[18, 19] Additionally, in contrast to the situation with creatinine, there is currently no certified reference measurement procedure for cystatin C to definitively establish target values for the reference material.[22]

Attention to accuracy of standardisation of cystatin C assays has important clinical and research implications. This was illustrated in a UK primary care study of older patients with stage 3 CKD, where use of the Abbott cystatin C assay classified a greater proportion as having more advanced CKD than use of $\text{GFR}_{\text{creatinine}}$, with associated increased monitoring costs, leading the authors to refute the recommendation of the NICE 2014 guideline in relation to use of cystatin C.[23]

Supplementary Tables

Supplementary Table 1. Linear and Deming regression analysis of the relationship between the Abbott and Siemens cystatin C assays.

	Linear Regression			Deming Regression		
	Estimate	95% CI	p	Estimate	95% CI	p
**Abbot Cystatin C**	0.94	0.92, 0.96	<0.001	0.95	0.92, 0.97	<0.001
**Constant**	-0.08	-0.12, -0.03	<0.001	-0.09	-0.13, -0.06	<0.001

Supplementary Table 2. Performance of cystatin C–containing GFR estimating equations compared to measured GFR before (lower row, italics) and after (upper row, not italicized) recalibration. Median measured GFR was 47.0 mL/min/1.73 m^2.

**Equation**	**Estimated GFR, median (IQR), mL/min/1.73 m^2**	**Bias (estimated minus measured GFR), mean difference (SD)[95% CI], mL/min/1.73 m^2**	**Bias (estimated minus measured GFR), median difference (IQR), mL/min/1.73 m^2**	**Accuracy, percentage of estimates within 30% of measured GFR (P_{30}) (95% CI)**
CKD-EPI$_{cystatin}$	42.3 [33.8, 53.4]	-3.4 (9.1) [-3.9, -2.9]	-4.1 (-9.3, 1.5)	89.5 (87.6, 91.2)
	36.4 [29.4, 45.5]	*-9.9 (7.9) [-10.3, -9.4]*	*-9.8 (-14.9, -5.3)*	*72.5 (69.8, 75.0)*
CKD-EPI$_{creatinine\text{-}cystatin}$	42.7 [34.6, 52.4]	-3.7 (7.3) [-4.1, -3.3]	-3.9 (-8.4, 1.1)	94.9 (93.5, 96.1)
	39.4 [32.2, 48.1]	*-7.2 (7.1) [-7.6, -6.8]*	*-7.2 (-11.7, -2.4)*	*90.4 (88.6, 92.0)*
CKD-EPI(2021)$_{creatinine\text{-}cystatin}$	45.2 [36.7, 55.4]	-1.1 (7.6) [-1.5, -0.6]	-1.3 (-6.1, 3.7)	94.9 (93.4, 96.1)
	41.4 [33.8, 50.3]	*-5.2 (7.2) [-5.6, -4.8]*	*-5.3 (-9.9, -0.4)*	*94.2 (92.7, 95.4)*
EKFC$_{cystatin}$	46.1 [37.9, 56.6]	0.1 (8.7) [-0.4, 0.6]	-0.4 (-5.5, 5.4)	91.0 (89.2, 92.6)
	40.6 [33.7, 49.4]	*-5.9 (8.0) [-6.3, -5.4]*	*-5.8 (-11.0, -0.80)*	*90.3 (88.5, 92.0)*
EKFC$_{creatinine\text{-}cystatin}$	44.6 [37.3, 53.4]	-2.1 (7.3) [-2.5, -1.7]	-2.1 (-6.8, 2.6)	94.9 (93.4, 96.1)
	41.7 [35.0, 49.8]	*-5.1 (7.3) [-5.5, -4.7]*	*-5.0 (-9.7, -0.3)*	*95.1 (93.7, 96.3)*

Supplementary Table 3. Performance of GFR estimating equations compared to the 2009/2012 CKD-EPI equations.

Test B	Test A		
	CKD-EPI$_{creatinine}$	CKD-EPI$_{cystatin}$	CKD-EPI$_{creatinine-cystatin}$
CKD-EPI$_{creatinine}$			
CKD-EPI$_{cystatin}$	-0.8 (-3.3, 1.7) p=0.5783		
CKD-EPI$_{creatinine-cystatin}$	**4.7 (2.9, 6.5) p<0.0001**	**5.5 (3.8, 7.2) p<0.0001**	
CKD-EPI(2021)$_{creatinine}$	-2.2 (-3.8, -0.7) p=0.0043	-1.5 (-4.1, 1.2) p=0.2945	-6.9 (-9.0, -4.9) p<0.0001
CKD-EPI(2021)$_{creatinine-cystatin}$	**4.6 (2.8, 6.4) p<0.0001**	**5.4 (3.6, 7.2) p<0.0001**	-0.1 (-1.2, 1.0) p>0.9999
EKFC$_{creatinine}$	-0.9 (-2.0, 0.3) p=0.1539	-0.1 (-2.6, 2.5) p>0.9999	-5.6 (-7.5, -3.7) p<0.0001
EKFC$_{cystatin}$	0.8 (-1.5, 3.1) p=0.5455	1.5 (-0.3, 3.4) p=0.1141	-3.9 (-5.7, -2.2) p<0.0001
EKFC$_{creatinine-cystatin}$	**4.6 (3.0, 6.3) p<0.0001**	**5.4 (3.4, 7.4) p<0.0001**	-0.1 (-1.4, 1.2) p>0.9999

The table shows % difference (95% CI) for P30 test B – P30 test A together with a P-value (positive values indicate test B superior to test A and negative values indicate test B inferior to test A). Significant positive differences shown in bold. McNemar’s test was used to compare P30 values of the equations against each other.

Supplementary Table 4. Performance of GFR estimating equations compared to measured GFR stratified by age, sex, diabetes, albuminuria, body mass index (BMI) and level of measured GFR. Results represent accuracy, percentage of estimates within 30% of measured GFR (P30 (95% CI)).

**Equation**	**Category**				
	**Age (years)**<br>**<50 (n=149)**	**50-59 (n=177)**	**60-69 (n=376)**	**70-79 (n=368)**	**≥80 (n=97)**
CKD-EPI$_{creatinine}$	87.9 (81.6, 92.7)	88.7 (83.1, 93.0)	88.8 (85.2, 91.8)	92.9 (89.8, 95.3)	91.8 (84.4, 96.4)
CKD-EPI$_{cystatin}$	90.6 (84.7, 94.8)	92.1 (87.1, 95.6)	87.0 (83.1, 90.2)	90.5 (87.0, 93.3)	88.7 (80.6, 94.2)
CKD-EPI$_{creatinine-cystatin}$	97.3 (93.3, 99.3)	94.4 (89.9, 97.3)	93.6 (90.7, 95.9)	95.7 (93.0, 97.5)	94.8 (88.4, 98.3)
CKD-EPI(2021)$_{creatinine}$	85.2 (78.5, 90.5)	89.3 (83.7, 93.4)	88.3 (84.6, 91.4)	88.6 (84.9, 91.6)	86.6 (78.2, 92.7)
CKD-EPI(2021)$_{creatinine-cystatin}$	93.3 (88.0, 96.7)	94.4 (89.9, 97.3)	94.4 (91.6, 96.5)	95.9 (93.4, 97.7)	95.9 (89.8, 98.9)
EKFC$_{creatinine}$	87.2 (80.8, 92.1)	88.7 (83.1, 93.0)	87.5 (83.7, 90.7)	91.8 (88.6, 94.4)	91.8 (84.4, 96.4)
EKFC$_{cystatin}$	82.6 (75.5, 88.3)	87.6 (81.8, 92.0)	92.0 (88.8, 94.6)	94.8 (92.1, 96.9)	91.8 (84.4, 96.4)
EKFC$_{creatinine-cystatin}$	90.6 (84.7, 94.8)	94.9 (90.6, 97.6)	94.4 (91.6, 96.5)	96.2 (93.7, 97.9)	97.9 (92.7, 99.7)
	**Gender**<br>**Males (n=680)**	**Females (n=487)**			
CKD-EPI$_{creatinine}$	88.5 (85.9, 90.8)	92.6 (89.9, 94.8)			
CKD-EPI$_{cystatin}$	90.3 (87.8, 92.4)	88.3 (85.1, 91.0)			
CKD-EPI$_{creatinine-cystatin}$	94.4 (92.4, 96.0)	95.7 (93.5, 97.3)			
CKD-EPI(2021)$_{creatinine}$	87.2 (84.5, 89.6)	89.1 (86.0, 91.7)			
CKD-EPI(2021)$_{creatinine-cystatin}$	94.4 (92.4, 96.0)	95.5 (93.2, 97.1)			
EKFC$_{creatinine}$	87.2 (84.5, 89.6)	92.4 (89.7, 94.6)			
EKFC$_{cystatin}$	93.7 (91.6, 95.4)	87.3 (84.0, 90.1)			
EKFC$_{creatinine-cystatin}$	95.1 (93.3, 96.6)	94.5 (92.0, 96.3)			
	**Diabetes**<br>**Not-diabetes (n=843)**	**Diabetes (n=324)**			
CKD-EPI$_{creatinine}$	90.7 (88.6, 92.6)	88.9 (85.0, 92.1)			
CKD-EPI$_{cystatin}$	89.9 (87.7, 91.9)	88.3 (84.4, 91.6)			
CKD-EPI$_{creatinine-cystatin}$	95.1 (93.5, 96.5)	94.4 (91.4, 96.7)			
CKD-EPI(2021)$_{creatinine}$	88.1 (85.8, 90.2)	87.7 (83.6, 91.0)			

CKD-EPI(2021)$_{creatinine\text{-}cystatin}$	94.7 (92.9, 96.1)	95.4 (92.5, 97.4)
EKFC$_{creatinine}$	90.6 (88.5, 92.5)	86.1 (81.9, 89.7)
EKFC$_{cystatin}$	90.3 (88.1, 92.2)	92.9 (89.5, 95.4)
EKFC$_{creatinine\text{-}cystatin}$	94.9 (93.2, 96.3)	94.8 (91.7, 96.9)

	**Albuminuria category (mg/mmol)**		
	**A0, <3 (n=483)**	**A1, 3.0-29.9 (n=396)**	**A2, ≥30 (n=269)**
CKD-EPI$_{creatinine}$	91.7 (88.9, 94.0)	90.4 (87.1, 93.1)	87.7 (83.2, 91.4)
CKD-EPI$_{cystatin}$	91.9 (89.1, 94.2)	86.9 (83.1, 90.0)	89.2 (84.9, 92.7)
CKD-EPI$_{creatinine\text{-}cystatin}$	95.7 (93.4, 97.3)	95.5 (92.9, 97.3)	92.9 (89.2, 95.7)
CKD-EPI(2021)$_{creatinine}$	89.6 (86.6, 92.2)	87.6 (84.0, 90.7)	86.2 (81.5, 90.1)
CKD-EPI(2021)$_{creatinine\text{-}cystatin}$	95.7 (93.4, 97.3)	95.2 (92.6, 97.1)	92.9 (89.2, 95.7)
EKFC$_{creatinine}$	89.9 (86.8, 92.4)	89.4 (85.9, 92.2)	88.5 (84.0, 92.0)
EKFC$_{cystatin}$	91.9 (89.1, 94.2)	90.2 (86.8, 92.9)	90.3 (86.2, 93.6)
EKFC$_{creatinine\text{-}cystatin}$	95.2 (92.9, 97.0)	94.7 (92.0, 96.7)	94.4 (91.0, 96.8)

	**BMI (kg/m^2)**	
	**<30 (n=668)**	**≥30 (n=499)**
CKD-EPI$_{creatinine}$	91.2 (88.8, 93.2)	89.0 (85.9, 91.6)
CKD-EPI$_{cystatin}$	90.9 (88.4, 92.9)	87.6 (84.4, 90.3)
CKD-EPI$_{creatinine\text{-}cystatin}$	96.0 (94.2, 97.3)	93.6 (91.1, 95.6)
CKD-EPI(2021)$_{creatinine}$	88.2 (85.5, 90.5)	87.8 (84.6, 90.5)
CKD-EPI(2021)$_{creatinine\text{-}cystatin}$	95.1 (93.1, 96.6)	94.6 (92.2, 96.4)
EKFC$_{creatinine}$	90.6 (88.1, 92.7)	87.8 (84.6, 90.5)
EKFC$_{cystatin}$	89.7 (87.1, 91.9)	92.8 (90.2, 94.9)
EKFC$_{creatinine\text{-}cystatin}$	94.9 (93.0, 96.4)	94.8 (92.5, 96.6)

	**Measured GFR (mL/min/1.73 m^2)**	
	**<45 (n=504)**	**≥45 (n=663)**
CKD-EPI$_{creatinine}$	88.1 (84.9, 90.8)	91.9 (89.5, 93.8)
CKD-EPI$_{cystatin}$	87.7 (84.5, 90.4)	90.8 (88.3, 92.9)
CKD-EPI$_{creatinine\text{-}cystatin}$	93.7 (91.2, 95.6)	95.9 (94.1, 97.3)
CKD-EPI(2021)$_{creatinine}$	81.7 (78.1, 85.0)	92.8 (90.5, 94.6)
CKD-EPI(2021)$_{creatinine\text{-}cystatin}$	92.7 (90.0, 94.8)	96.5 (94.8, 97.8)

$EKFC_{creatinine}$	88.9 (85.8, 91.5)	89.7 (87.2, 91.9)
$EKFC_{cystatin}$	87.9 (84.7, 90.6)	93.4 (91.2, 95.1)
$EKFC_{creatinine-cystatin}$	92.5 (89.8, 94.6)	96.7 (95.0, 97.9)

Supplementary Table 5. Performance of CKD-EPI and EKFC GFR estimating equations compared to measured GFR in participants according to ethnicity.

Equation	Reported ethnicity[a]				Difference (95% CI) for black people with and without adjustment factor; p-values (n=60)[b]
	White (n=1014)	South-Asian (n=66)	Black (n=60)	Black (n=60), adjustment factor removed	
CKD-EPI$_{creatinine}$	90.8 (88.9, 92.5)	86.4 (75.7, 93.6)	81.7 (69.6, 90.5)	70.0 (56.8, 81.2)	-11.7 (-23.8, 0.4); p=0.0654
CKD-EPI$_{cystatin}$	89.6 (87.6, 91.5)	86.4 (75.7, 93.6)	90.0 (79.5, 96.2)	N/A	N/A
CKD-EPI$_{creatinine-cystatin}$	95.0 (93.4, 96.2)	93.9 (85.2, 98.3)	95.0 (86.1, 99.0)	90.0 (79.5, 96.2)	-5.0 (-12.2, 2.2); p=0.2500
CKD-EPI(2021)$_{creatinine}$	88.9 (86.8, 90.7)	84.8 (73.9, 92.5)	75.0 (62.1, 85.3)	N/A	N/A
CKD-EPI(2021)$_{creatinine-cystatin}$	95.1 (93.6, 96.3)	92.4 (83.2, 97.5)	93.3 (83.8, 98.2)	N/A	N/A
EKFC$_{creatinine}$	90.2 (88.2, 92.0)	84.8 (73.9, 92.5)	76.7 (64.0, 86.6)	N/A	N/A
EKFC$_{cystatin}$	91.9 (90.1, 93.5)	83.3 (72.1, 91.4)	85.0 (73.4, 92.9)	N/A	N/A
EKFC$_{creatinine-cystatin}$	95.5 (94.0, 96.7)	89.4 (79.4, 95.6)	91.7 (81.6, 97.2)	N/A	N/A

Results represent accuracy, percentage of estimates within 30% of measured GFR (P30 (95% CI)). N/A, not applicable: the original version of these equations did not contain an African-Caribbean adjustment factor.

[a]Ethnicity data were unavailable in 3 individuals and 24 individuals were of non-White, South-Asian or Black ethnicity. Twenty seven individuals were therefore excluded from this analysis

[b]McNemar test was used

Legend for Supplementary Figure 1. Flow of participants through the study.

Legend for Supplementary Figure 2. Scatter plot comparing Abbott and Siemens cystatin C measurements. The solid red line shows the linear regression line of best fit and the dotted line shows the line of identity.

Legend for Supplementary Figure 3. Box and whisker plot showing bias of CKD-EPI(2021) estimated GFR equations compared to measured GFR.

Footnote for Supplementary Figure 3. Data for the CKD-EPI(2021) estimated GFR equations are shown as box and whisker plots. The box shows the median and the first (Q1) and third quartiles (Q3). The whiskers span all data points within 1.5 IQR of the nearer quartile, with Tukey outliers outside of this range (<Q1-1.5IQR or >Q3+1.5IQR).

Legend for Supplementary Figure 4. Lowess plot showing bias of CKD-EPI(2021) estimated GFRs (y axes) compared to measured GFR (mGFR, x axes).

Footnote for Supplementary Figure 4. Individual plots depict bias of CKD-EPI(2021)$_{creatinine}$ (left panel) and CKD-EPI$_{creatinine-cystatin}$ (right panel) estimated GFRs against measured GFR. Units of measurement are mL/min/1.73 m^2 in all cases. Bias plots are shown with lowess (locally weighted scatterplot smoothing) function (black curvilinear line). The horizontal black line shows zero bias and the dashed black line shows mean bias.

Supplementary References

1. Carter JL, Stevens PE, Irving JE, et al. Estimating glomerular filtration rate: comparison of the CKD-EPI and MDRD equations in a large UK cohort with particular emphasis on the effect of age. *QJM* 2011;104(10):839-47.
2. Major RW, Shepherd D, Medcalf JF, et al. Comorbidities and outcomes in South Asian individuals with chronic kidney disease: an observational primary care cohort. *Nephrol Dial Transplant* 2021;37(1):108-14. doi: 10.1093/ndt/gfaa291 [published Online First: 2021/01/14]
3. Levey AS, Coresh J, Greene T, et al. Using standardized serum creatinine values in the modification of diet in renal disease study equation for estimating glomerular filtration rate. *Ann Intern Med* 2006;145(4):247-54.
4. Turin TC, Tonelli M, Manns BJ, et al. Chronic kidney disease and life expectancy. *Nephrol Dial Transplant* 2012;27(8):3182-6. doi: 10.1093/ndt/gfs052 [published Online First: 2012/03/24]
5. Bellomo R, Ronco C, Kellum JA, et al. Acute renal failure - definition, outcome measures, animal models, fluid therapy and information technology needs: the Second International Consensus Conference of the Acute Dialysis Quality Initiative (ADQI) Group. *Crit Care* 2004;8(4):R204-12.
6. Rowe C, Sitch AJ, Barratt J, et al. Biological variation of measured and estimated glomerular filtration rate in patients with chronic kidney disease. *Kidney Int* 2019;96(2):429-35. doi: 10.1016/j.kint.2019.02.021 [published Online First: 2019/05/16]
7. Brochner-Mortensen J. A simple method for the determination of glomerular filtration rate. *Scand J Clin Lab Invest* 1972;30(3):271-4.
8. Levey AS, Stevens LA, Schmid CH, et al. A new equation to estimate glomerular filtration rate. *Ann Intern Med* 2009;150(9):604-12.
9. Inker LA, Schmid CH, Tighiouart H, et al. Estimating glomerular filtration rate from serum creatinine and cystatin C. *N Engl J Med* 2012;367(1):20-9. doi: 10.1056/NEJMoa1114248 [published Online First: 2012/07/06]
10. Pottel H, Bjork J, Courbebaisse M, et al. Development and Validation of a Modified Full Age Spectrum Creatinine-Based Equation to Estimate Glomerular Filtration Rate : A Cross-sectional Analysis of Pooled Data. *Ann Intern Med* 2021;174(2):183-91. doi: 10.7326/M20-4366 [published Online First: 2020/11/10]
11. Pottel H, Bjork J, Rule AD, et al. Cystatin C-Based Equation to Estimate GFR without the Inclusion of Race and Sex. *N Engl J Med* 2023;388(4):333-43. doi: 10.1056/NEJMoa2203769 [published Online First: 2023/02/01]
12. Inker LA, Eneanya ND, Coresh J, et al. New Creatinine- and Cystatin C-Based Equations to Estimate GFR without Race. *N Engl J Med* 2021;385(19):1737-49. doi: 10.1056/NEJMoa2102953 [published Online First: 2021/09/24]
13. Greenberg N, Roberts WL, Bachmann LM, et al. Specificity characteristics of 7 commercial creatinine measurement procedures by enzymatic and jaffe method principles. *Clin Chem* 2012;58(2):391-401.
14. Blake GM, Roe D, Lazarus CR. Long-term precision of glomerular filtration rate measurements using 51Cr-EDTA plasma clearance. *Nucl Med Commun* 1997;18(8):776-84.
15. Burniston M. Clinical guideline for the measurement of glomerular filtration rate (GFR) using plasma sampling. Approved by the British Nuclear Medicine Society Professional Standards Committee, 2018.
16. Du Bois E, Du Bois D. A formula to estimate the approximate surface area if height and weight be known. *Arch Intern Med* 1916;17:863-71.
17. Grubb A, Blirup-Jensen S, Lindstrom V, et al. First certified reference material for cystatin C in human serum ERM-DA471/IFCC. *Clin Chem Lab Med* 2010;48(11):1619-21.

18. Eckfeldt JH, Karger AB, Miller WG, et al. Performance in Measurement of Serum Cystatin C by Laboratories Participating in the College of American Pathologists 2014 CYS Survey. *Arch Pathol Lab Med* 2015;139(7):888-93. doi: 10.5858/arpa.2014-0427-CP [published Online First: 2015/04/18]
19. Bargnoux AS, Pieroni L, Cristol JP, et al. Multicenter Evaluation of Cystatin C Measurement after Assay Standardization. *Clin Chem* 2017;63(4):833-41. doi: 10.1373/clinchem.2016.264325 [published Online First: 2017/02/12]
20. Kilbride HS, Stevens PE, Eaglestone G, et al. Accuracy of the MDRD (Modification of Diet in Renal Disease) study and CKD-EPI (CKD Epidemiology Collaboration) equations for estimation of GFR in the elderly. *Am J Kidney Dis* 2013;61(1):57-66.
21. O'Seaghdha CM, Tin A, Yang Q, et al. Association of a cystatin C gene variant with cystatin C levels, CKD, and risk of incident cardiovascular disease and mortality. *Am J Kidney Dis* 2014;63(1):16-22.
22. Karger AB, Long T, Inker LA, et al. Improved Performance in Measurement of Serum Cystatin C by Laboratories Participating in the College of American Pathologists' 2019 CYS Survey. *Arch Pathol Lab Med* 2022 doi: 10.5858/arpa.2021-0306-CP [published Online First: 2022/02/23]
23. Shardlow A, McIntyre NJ, Fraser SDS, et al. The clinical utility and cost impact of cystatin C measurement in the diagnosis and management of chronic kidney disease: A primary care cohort study. *PLoS Med* 2017;14(10):e1002400. doi: 10.1371/journal.pmed.1002400 [published Online First: 2017/10/11]
