# Supplementary material for: Test accuracy of glomerular filtration rate estimation with creatinine and cystatin C in adults with moderate chronic kidney disease: prospective cohort study
Source: BMJ Med. 2026 Jan 21;5(1):e001827. doi: 10.1136/bmjmed-2025-001827 (PMC12829397; doi:10.1136/bmjmed-2025-001827)
Supplement: Supplementary file 6 [file bmjmed-2025-001827.reviewer_comments.pdf]

PEER REVIEW HISTORY

BMJ Medicine publishes all reviews undertaken for accepted manuscripts. Reviewers are asked to complete a checklist review form and are provided with free text boxes to elaborate on their assessment. These free text comments are reproduced below.

This paper was submitted to another journal from BMJ but declined for publication following peer review. The authors addressed the reviewers' comments and submitted the revised paper to BMJ Medicine. The paper was subsequently accepted for publication at BMJ Medicine.

ARTICLE DETAILS

**TITLE (PROVISIONAL)**	Glomerular filtration rate estimation using creatinine and cystatin C: a prospective study of test accuracy in adults with moderate chronic kidney disease
**AUTHORS**	Lamb, Edmund; Barratt, Jonathan; Brettell, Elizabeth; Cockwell, Paul; Dalton, R; Deeks, Jonathan; Eaglestone, Gillian; Kalra, Philip; Khunti, Kamlesh; Loud, Fiona; Mead, Phoebe; Ottridge, Ryan; Pellatt-Higgins, Tracy; Potter, Aisling; rowe, ceri; Scandrett, Katie; Sharpe, Claire; Shinkins, Bethany; Sitch, Alice; Smith, Alison; Stevens, Paul; Sutton, Andrew J; Taal, Maarten

VERSION 1 - REVIEW

**REVIEWER NAME**	Reviewer 1
**REVIEWER AFFILIATION**	
**REVIEWER CONFLICT OF INTEREST**	None
**DATE REVIEW RETURNED**	04-Jun-2025

**GENERAL COMMENTS**	1. General Comments<br>This is a well-executed, prospective diagnostic accuracy study evaluating the performance of various GFR estimating equations against iohexol-measured GFR in a real-world clinical population with moderate CKD. The authors should be commended for the methodological rigor, the size of the cohort, and the thoroughness of the comparative analysis involving both creatinine-based, cystatin C–based, and dual-marker equations.<br>However, several important areas require clarification and improvement to strengthen the manuscript's clarity, transparency, and clinical relevance. These points include the clarity of study objectives, the framing of estimation performance, the interpretation of subgroup variability, and the absence of discussion around complementary clinical markers like albuminuria.<br><br>2. Major Comments<br>1. Clarity of Study Objective and Framing in Introduction<br>While the study focuses on comparing the accuracy of existing GFR estimating equations, this is not explicitly stated in the Introduction. The current framing—particularly in the final paragraph—could mislead readers into thinking that a new estimating equation is being proposed or derived. The distinction between validating existing equations and developing new ones should be made more clear, especially in the final paragraph of the Introduction. Additionally, the Methods section should reinforce this positioning explicitly.

	2. Inclusion Criteria and Risk of Selection Bias<br>The study uses MDRD-based eGFR values (30–59 ml/min/1.73m²) as the inclusion criterion. Since MDRD is a creatinine-only equation with well-documented bias, especially in individuals with atypical muscle mass or across racial groups, this approach may introduce selection bias. The resulting study cohort may disproportionately include individuals misclassified by MDRD, which could influence the apparent performance of other equations (particularly dual-marker equations that correct for such misclassifications). The authors should explicitly discuss this potential source of bias and, if possible, consider sensitivity analyses using measured GFR ranges.<br>3. Interpretation of Table 3 and Scientific Novelty<br>Table 3 shows that dual-marker equations generally outperform creatinine-only or cystatin C–only equations, especially in achieving P30 >90%. However, from a methodological standpoint, this is an expected outcome: adding more variables generally improves model accuracy. The Discussion should reflect that the superior performance of dual equations is not necessarily a novel scientific finding, but a predictable statistical result. What is clinically relevant, however, is how and when such dual testing should be used. We recommend that the authors provide a clearer interpretation of their findings in terms of practical implications, including clinical contexts or subpopulations where dual-marker testing may be especially warranted.<br>4. Discussion of Subgroup Performance (Supplementary Table 3)<br>While the main text highlights the overall P30 performance of several equations, Supplementary Table 3 shows significant variation in performance across subgroups (e.g., by age, sex, and ethnicity). For example, even the dual-marker equations exhibit reduced accuracy in older adults and Asian individuals. These findings are not adequately addressed in the Discussion. We recommend that the authors acknowledge this heterogeneity and avoid overgeneralising the applicability of dual-marker equations.<br>5. Role of Albuminuria Not Acknowledged<br>The manuscript provides a thoughtful reflection on the limits of estimation based on creatinine and cystatin C, noting that biological and analytical variability set an upper limit on achievable accuracy. However, it omits any mention of albuminuria, which is a routinely used complementary marker in CKD diagnosis and risk stratification per KDIGO guidelines. Briefly acknowledging that eGFR is only one part of CKD staging, and that albuminuria provides complementary information, would help contextualise the limitations of the study and ground its findings in routine clinical practice.<br>3. Minor Comments and Editorial Suggestions<br>In the Supplementary Methods section, the reported P30 value for the BIS1creatinine equation is listed as '94.9 (82.3, 87.3)'. This appears to be a typographical error, as the reported 95% confidence interval does not include the point estimate (94.9), and the lower bound (82.3) is higher than the upper bound (87.3), which is not logically possible. Please review and correct this value to ensure consistency and accuracy of the supplementary data.<br>The term 'accuracy' is used frequently, but it would be helpful to define this more precisely in relation to P30, bias, and RMSE at the beginning of the Results or Methods section.<br>Please ensure that the manuscript is consistent in whether it refers to 'CKD-EPI 2021' or 'new CKD-EPI' and 'original CKD-EPI' versus '2009 CKD-EPI' to avoid confusion.

**REVIEWER NAME**	Shlipak, Michael

**REVIEWER AFFILIATION**	UCSF
**REVIEWER CONFLICT OF INTEREST**	I have served on advisory boards for Astra Zeneca and Boehringer Ingelheim; research funds to my insitution from Bayer that support my research team.
**DATE REVIEW RETURNED**	04-Jun-2025

**GENERAL COMMENTS**	This is a companion paper to Scandrett et al, which I apparently read out of sequence. Having reviewed the longitudinal GFR paper, I was asked to review this cross-sectional paper. Several of my overall comments about data presentation are reflected in the other review – specifics to follow. However, whereas I viewed the longitudinal paper as being a highly novel contribution to the literature, this cross-sectional manuscript from the same cohort has less novelty and more limitations in its presentation. The overall design of a 6-center study of measured GFR among persons with CKD remains an excellent achievement. However, several limitations of this cross-sectional study limit its impact and make me question the validity of its conclusions.<br>Summary box: the topic summary includes two issues that I found inappropriate.<br>The relative costs of creatinine and cystatin C. I also practice medicine in a national healthcare system with constrained costs (U.S. Veterans Health), and we are told the actual lab tests for all tests at our hospital. As with this research study, we use enzymatic creatinine, and the cost is $1.50/test. Our cystatin C at moderately high volume is $2.75. For reference, our 25(OH) vitamin D is $10, our PTH is $28, and our NtproBNP is $50. The narrative around the costs of cystatin C is suppressive and beyond that used for any other lab test that I have read literature about. The authors quote relative costs in pounds of 0.43 for Cr (is that enzymatic or Jaffe?) and 3.8 for cysC. Even at this 10-fold quoted cost, nothing about this paper would lead to 50 million cysC tests being done in the UK – the reported number for Cr.<br><br>Subgroups of black and south Asians: the authors were sadly only able to recruit 5% (n=60) each for these two groups. These low numbers warrant noting in the text, but they are far too low for the sweeping conclusions cited in the summary, the abstract, and the discussion. The diverse recruitment for this study was a near-complete failure with about 90% white participants. The fact that diverse recruitment was attempted does not mitigate this fact, and it suggests that the recruited subset may have substantial bias from their representative communities.<br><br>The need to calibrate cystatin C values: I appreciate the struggle that the authors went through upon learning that the Abbott platform leads to biased cysC values; this is something we learned at the UCSF university hospital in 2020. Calibration is an inexact process. For the longitudinal paper, I think this is less of an issue because changes in the measure are unlikely to be affected by the calibration bias. However, I believe that the authors should re-measure all cystatin C values at baseline for this paper using an optimal method (Siemens perhaps). At 3.8 pounds/test * 1167, this would not have costed too much relative to the massive investment in this study to date. The calibration exercise substantially lowers the confidence an informed reader can have in the results.<br><br>Choice of a cohort with eGFRcr 30-60: This seemed appropriate for the longitudinal paper, as these are the patients who require

	monitoring. However, this creates biases for the study that make Cr look both worse and better. Although mentioned in the discussion, the bias is substantial. A) Unfair to Cr – the results state that Cr has more bias at the low and high values of mGFR; this is self-evident because mGFR can range below and above the 30-60 interval and eGFRcr cannot. This is a study limitation not a research finding. B) Cr looks better – by definition, all participants have recognized CKD, and 60% have substantial urine albumin suggesting they may be recruited from nephrology clinic. Creatinine's major weakness is its insensitivity which increases with age and frailty. So, Cr has a relative advantage in this study that is a major bias compared with an unselected population.<br><br>Results presentation:<br>Figure 1 – I found these 15 panels to be unreadable. Maybe 2 panels would work or spread across multiple pages.<br>Table 2 – a) as in my review of the other paper, the main findings should show only 6 equations – Cr, cys, both for 2 equation families. The race or no-race issue cannot be decided in this study given 60 black persons. All other equations can be parked in a supplement.<br>Table 3 – The key findings here are that the combined equations are all 5% higher on the P30 scale compared with the Cr referent equation. I still believe a figure could summarize these results, limiting to the top 6 equations with others in supplement.<br>Table 4 – these data have the small black and south Asian groups. Its fine to show the data, but these are basically pilot data. A statement was made about significance for the Black subgroup with race-free equation; this did not seem appropriate as based on the white distribution and not compared with the paired results from the data with race included. The absolute difference of 5% is the powered minimum detectable difference for the entire cohort of 1000+.<br>Supplementary Table 3 – this table is overly dense, but these data on varied performance by subgroups of age, sex, are very important and could be presented in the main paper in a condense format. Again, figures are efficient for overviews of lots of data.<br>Discussion – As already stated I think the conclusion about race subgroups are inappropriately based on very small numbers. For the South Asian subset, for example, it might be informative to cite a paper by my colleagues from the UK Biobank among 7,738 South Asians, albeit without mGFR (Chen DC JAHA, 2023) https://pubmed.ncbi.nlm.nih.gov/36695320/.<br>The authors make important points about the challenges of an imprecise reference standard, mGFR, and the limited ability of biomarkers to exceed the precisions described in this paper. Regarding cysC laboratory methods, the authors cite studies from 2017, whereas papers by Amy Karger suggest that the measurement and standardization are improving.

**REVIEWER NAME**	Reviewer 3
**REVIEWER AFFILIATION**	
**REVIEWER CONFLICT OF INTEREST**	None
**DATE REVIEW RETURNED**	04-Jun-2025

**GENERAL COMMENTS**	None

**REVIEWER NAME**	Liu, Dandan
**REVIEWER AFFILIATION**	Beijing Shijitan Hospital, Capital Medical University
**REVIEWER CONFLICT OF INTEREST**	None
**DATE REVIEW RETURNED**	04-Jun-2025

**GENERAL COMMENTS**	Thank you for the opportunity to provide review for this well-written paper.<br>Originality - does the work add enough to what is already in the published literature? If so, what does it add?<br>The article assess the accuracy of GFR estimating equations, including those utilizing cystatin C, in a representative English population comprising adults with stage 3 CKD (MDRD creatinine-estimated GFR of 30-59 mL/min/1.73 m2, sustained for at least 3 months prior to recruitment). The study includes black and South Asian individuals from six UK centers. At present, there are no large -scale multi-center studies comparing estimating equations with measured GFR. The article is innovative and provides high-level evidence for addressing urgent problems in current clinical practice.<br>Importance of work to readers - does this work matter to researchers, clinicians, patients, teachers, or policymakers?<br>The staging of CKD is primarily based on GFR. Measured GFR values are impractical for routine clinical use and GFR is typically estimated using established equations. Different CKD stages require distinct treatment strategies. An inaccurate GFR estimation can lead to completely different treatment measures, and result in poor prognoses for patients near a stage threshold. Therefore, an accurate GFR estimation through validated equations is crucial for precise CKD staging. The findings in this article have significant implications for researchers, clinicians, patients, teachers and policymakers alike.<br>Evidence-based medicine - is the evidence presented scientifically and reliable?<br>The research data were obtained from six centers, featuring a reasonable sample size, an acceptable dropout rate, and a well-balanced baseline distribution. The statistical analysis methods were appropriate, and the evidence presented were both scientifically valid and reliable.<br>Research question - is it clearly defined and appropriately answered?<br>It was clearly defined and appropriately answered.<br>Study design - is the overall design adequate?<br>The overall design of study was appropriate and adequate to answer the research question.<br>Participants - are they adequately described and their conditions well defined?<br>In the supplementary file(P32), participants were adequately described, the conditions and both interventions were clearly defined, inclusion and exclusion criteria were explicitly stated and a rationale was given for these decisions.<br>Methods - are they adequately described? Are they ethical?<br>The methodology was described in this article and previously published protocol, enabling readers to access comprehensive information. This study adhered to the fundamental principles of ethics.<br>Results - do they answer the research question? Are they credible and well-presented?<br>The results had answered the research question, demonstrating credibility and clarity in presentation. However, considering the

	topic's popularity and its potential to attract significant interest, minor revisions are needed to enhance expression. For instance, in Supplementary Figure 2, the Gantt chart, although the article has provided an explanation for the dropout cases, it would be beneficial to add arrow lines connecting the dropped-out subjects to the corresponding stages to make the logic becomes clearer.<br>Interpretation and conclusions - are they warranted by and sufficiently derived from/focused on the data? Is the message clear?<br>These conclusions were warranted by and sufficiently derived from/focused on the data. There were no distractions or irrelevant conclusions and the findings were discussed in the light of previous evidence. The study was elegantly presented and the message was clear. In particular, the author intends to analyze potential differences in estimated GFR using equations among different races, especially the black population. In the results section, 60 black individuals were actually enrolled. The author compared number of black cases obtained in this study with the proportion of the black population in the UK, and analyzed the results objectively. I recommend that the author revise the discussion section. Analyze and discuss in the order of :<br>1.All GFR estimates showed negative bias overall, with creatinine-based equations exhibiting a particularly pronounced shift in bias depending on level of GFR compared to reference measured GFR.<br>2.The inclusion of cystatin C within combined equations reduced bias overall and lessened, though did not entirely eliminate, the bias/GFR level effect. Several equations, especially the combined creatinine and cystatin equations, achieved clinically acceptable performance as defined by P30>90%.<br>3.Simple removing the black race adjustment factor from creatinine-based GFR equations may reduce accuracy.<br>The discussion should clear and concise, and avoid interweaving the analyses.<br>References - are they up to date and relevant? Any omissions?<br>Most of the references were current, appropriate for the content and highly relevant. While some references were not up to date, they remained representative and thus none of the references required omission.<br>Abstract/summary/key messages - do they reflect accurately what the paper says?<br>I recommend the “Abstract” needs to be revised.<br>1.The “Objective” should be revised to, for example: To compare the diagnosis validity of estimates of GFR equations to measured GFR.<br>2.The “Design” should be revised to: prospective, multi-center.<br>3.The “Participants” should be revised to: adults with moderate CKD (estimated GFR 30-59 mL/min/1.73 m2 sustained over at least 3 months prior to recruitment).<br>4.The “Summary” need no revision.<br>Reporting standards - does the paper comply with relevant standards? If the paper is a randomized controlled trial we will have asked the authors to provide the protocol and a CONSORT checklist. Other research designs should have the relevant checklist included (PRISMA, STARD, etc).<br>The article is not a randomized experiment and complies with relevant standards.

**REVIEWER NAME**	Kurth, Tobias
**REVIEWER AFFILIATION**	Charité, Berlin, Germany
**REVIEWER CONFLICT OF INTEREST**	TK reports having received research grants from the German Federal Joint Committee (G-BA) and personal compensation from

	the North-East German Society for Gynecological Oncology (NOGGO), AbbVie, Eli Lilly & Company, Novartis, the BMJ, and Frontiers.
**DATE REVIEW RETURNED**	04-Jun-2025

**GENERAL COMMENTS**	In this study of patients with stage III chronic kidney disease (CKD) from six primary, secondary, and tertiary care centers in England, Lamb and colleagues evaluated the accuracy and precision of 15 published equations for estimating glomerular filtration rate (GFR). A total of 1,167 adults with confirmed stage III CKD were included. Estimated GFR values were compared against measured GFR using iohexol clearance. The study has been registered and there is a considerable overlap with a publication in has been published in the National Institute of Health Research Journals Library. Accuracy was assessed using the P30 metric (percentage of estimates within 30% of the reference value), as recommended by the National Kidney Foundation–Kidney Disease Outcomes Quality Initiative. The findings showed a critical role of assay calibration for cystatin C measurements; following calibration adjustment, all equations achieved a P30 above 84.1%. Creatinine-based equations demonstrated variable bias depending on GFR level, while equations combining creatinine and cystatin C yielded higher accuracy than those based on a single biomarker. Differences in performance across subgroups defined by age, sex, diabetes status, albuminuria, body mass index, GFR, and ethnicity were equation-specific, with notably reduced accuracy among Black individuals when the race coefficient was omitted.<br><br>Given the widespread use of GFR estimating equations in clinical practice, this study offers valuable insights not only for general practitioners. The methodology is clearly described and appropriately executed, and the results are presented in a clear and accessible manner. Nonetheless, several study aspects could benefit from further clarification or refinement. The authors may want to consider the following aspects.<br><br>1. From both the main manuscript and the supplementary information, it remains unclear what the target population is and how the study sample reflects that population. One may infer that the target population comprises individuals with stage III CKD living in the catchment areas of the six participating centers (Birmingham, Canterbury, Derby, Leicester, Salford, and London [King's College Hospital]). However, it is not specified how many individuals with stage III CKD reside in these areas, how many were seen at the centers, how many were invited to participate, or what proportion ultimately enrolled. The flow chart shown in Supplementary Figure 2 does not help clarify these points. As such, the generalizability of the study findings remains uncertain.<br><br>2. Related to my previous point, I recommend that the authors refrain from describing the sample as "representative." It is unclear whether the participants constitute a representative sample of the catchment area populations or patients with stage III CKD more broadly. Moreover, the proportion of non-white participants was approximately 31% lower than reported in recent census data, a difference I would not consider "slight." It is also unclear whether the census figures refer specifically to the populations served by the six participating centers. This further limits claims of representativeness. The lower proportion of non-white participants

	may also reduce the statistical power for subgroup analyses within this population.<br><br>3. The authors should report the time frame during which participants were recruited. This information is difficult to find, even when looking the registered protocol or the study's website. The study website indicates that 1,249 participants were recruited over a 35-month period, which differs from the 1,229 participants reported in the submitted manuscript. This discrepancy should be clarified.<br><br>4. The authors should already state in the Introduction of the Methods section what the rationale was to use the P30 cut point. If I am not mistaken, this is only mentioned in the supplementary material.<br><br>5. The details in Figure 1 are difficult to interpret, even when zooming in to the maximum extent. The 15 individual panels within the figure are too small to be readable. I also recommend avoiding using red and green as contrasting colors (in all Figures), which may hinder interpretation for individuals with red-green color vision deficiency.<br><br>6. The authors should consider providing a rationale for selecting the covariates used for stratification in the explanatory analyses. Additionally, some continuous variables were dichotomized, which may obscure relevant information.<br><br>7. The titles of the Tables are pretty long. The authors may consider moving some information into the corresponding table footnotes to improve readability.<br><br>8. I do not see a conflict of interest, but the authors may want to reconsider the statement that support from pharmaceutical companies was unrelated to the study. Several of these companies provide diagnostic materials or medications used in managing CKD, and given that the study addresses diagnostic accuracy, this relationship may still be relevant.

**REVIEWER NAME**	Naesens, Maarten
**REVIEWER AFFILIATION**	KU Leuven
**REVIEWER CONFLICT OF INTEREST**	None
**DATE REVIEW RETURNED**	04-Jun-2025

**GENERAL COMMENTS**	The authors evaluate eGFR accuracy in adults patients with CKD, using 15 different published equations. The conclusion that the CKD -EPI formula with creatinine performs reasonably well (with measured iohexol clearance as ground truth), and that incorporating cystatin C has added value in this context, is relevant. The prospective study was performed in 1167 individuals recruited at 6 hospitals in the UK. Overall, this appears to be a solid and well-conducted study.<br><br>General Comments<br>- One could question the representativeness of the findings for clinical practice, as the study was conducted in what seems to be an idealized setting. I suspect that in real-world clinical environments, the differences between the equations may be even smaller.

	- The manuscript is somewhat difficult to follow due to a lack of clear structure and limited visual support. A more intuitive narrative and better use of figures would greatly enhance readability. Figure 1, for example, is currently unreadable and conceptually unclear. Moreover, plotting bias versus measured GFR is less intuitive than plotting estimated versus measured GFR, which would allow for a more direct assessment of accuracy. Including R^2 values in such plots would also help readers better understand the performance of the equations.<br>- Table 2 would benefit from visualization to make the data more accessible.<br>- Table 3 is dense with p-values, making it difficult to identify the key findings. While the differences in P30 scores are not incorrect, they are somewhat abstract and hard to interpret without additional context.<br>- The use of P30 as the primary accuracy metric stands out. While this is in line with NKF and KDIGO recommendations, it remains a somewhat arbitrary threshold. It is commendable that the authors also report RMSE in Table 2, which provides a more continuous measure of error. I would strongly recommend also including R^2 values and corresponding visualizations to complement the P30 and RMSE metrics.<br>- The limited representation of non-White individuals is a constraint, though this is acknowledged and appears to be a limitation of the cohort itself. The overall good performance of all equations may be partly due to the CKD stage 3 population. It is unclear why the recruitment was not extended to a broader population, which could have improved the generalizability of the findings.<br><br>Overall, this is a well-executed study that contributes to the growing literature on the best choice of eGFR formula. My comments above hopefully help improve the manuscript, especially for a general readership.

VERSION 1 – AUTHOR RESPONSE

Reviewer 1

1. General Comments
This is a well-executed, prospective diagnostic accuracy study evaluating the performance of various GFR estimating equations against iohexol-measured GFR in a real-world clinical population with moderate CKD. The authors should be commended for the methodological rigor, the size of the cohort, and the thoroughness of the comparative analysis involving both creatinine-based, cystatin C–based, and dual-marker equations.

Thank you for your supportive comments.
However, several important areas require clarification and improvement to strengthen the manuscript's clarity, transparency, and clinical relevance. These points include the clarity of study objectives, the framing of estimation performance, the interpretation of subgroup variability, and the absence of discussion around complementary clinical markers like albuminuria.

2. Major Comments
1. Clarity of Study Objective and Framing in Introduction
While the study focuses on comparing the accuracy of existing GFR estimating equations, this is not explicitly stated in the Introduction. The current framing—particularly in the final paragraph—could mislead readers into thinking that a new estimating equation is being proposed or derived. The

distinction between validating existing equations and developing new ones should be made more clear, especially in the final paragraph of the Introduction. Additionally, the Methods section should reinforce this positioning explicitly.

We have made it clearer in the abstract and introduction that we have studied published GFR equations. This was already stated in the first sentence of the methods section.

2. Inclusion Criteria and Risk of Selection Bias

The study uses MDRD-based eGFR values (30–59 ml/min/1.73m²) as the inclusion criterion. Since MDRD is a creatinine-only equation with well-documented bias, especially in individuals with atypical muscle mass or across racial groups, this approach may introduce selection bias. The resulting study cohort may disproportionately include individuals misclassified by MDRD, which could influence the apparent performance of other equations (particularly dual-marker equations that correct for such misclassifications). The authors should explicitly discuss this potential source of bias and, if possible, consider sensitivity analyses using measured GFR ranges.

MDRD-based eGFR values (30–59 mL/min/1.73m²) were used as the inclusion criterion primarily because this was standard clinical practice in England at the time of study commencement. We have now clarified the reason for using this equation for patient selection in the supplementary file (page 2). The MDRD equation has been reported to have a negative bias, causing over-diagnosis of CKD stage 3. However, in our data, nearly all creatinine-based GFR estimates were negatively biased compared to measured GFR, and to a similar degree, with MDRD giving slightly more negative estimates than some equations (e.g. CKD-EPI$_{creatinine}$) and less negatively biased estimates than others (e.g. EKFC$_{creatinine}$). We do not therefore believe that our results would have been very different whichever creatinine-based GFR estimate we had used for study recruitment. Furthermore, statistical comparisons within the study were of the GFR equations against measured GFR (as reference test), and of the GFR equations against CKD-EPI$_{creatinine}$ (as currently recommended for clinical use). The MDRD equation data and comparisons themselves have subsequently been removed from the paper in response to other reviewer comments.

3. Interpretation of Table 3 and Scientific Novelty

Table 3 shows that dual-marker equations generally outperform creatinine-only or cystatin C–only equations, especially in achieving P30 >90%. However, from a methodological standpoint, this is an expected outcome: adding more variables generally improves model accuracy. The Discussion should reflect that the superior performance of dual equations is not necessarily a novel scientific finding, but a predictable statistical result. What is clinically relevant, however, is how and when such dual testing should be used. We recommend that the authors provide a clearer interpretation of their findings in terms of practical implications, including clinical contexts or subpopulations where dual-marker testing may be especially warranted.

We do not agree that it is a universal truth that adding more markers improves equation accuracy. Adding beta trace protein and beta-2 microglobulin to cystatin C and creatinine equations for example did not improve accuracy (Inker et al, AJKD 2016). We have provided a clearer statement in the abstract conclusion as to when the combined equations should be used in preference, and this is also described elsewhere in the discussion (pages 12-13 and 15).

4. Discussion of Subgroup Performance (Supplementary Table 3)

While the main text highlights the overall P30 performance of several equations, Supplementary Table 3 shows significant variation in performance across subgroups (e.g., by age, sex, and ethnicity).

For example, even the dual-marker equations exhibit reduced accuracy in older adults and Asian individuals. These findings are not adequately addressed in the Discussion. We recommend that the authors acknowledge this heterogeneity and avoid overgeneralising the applicability of dual-marker equations.

We are unclear which data the reviewer is referring to. In terms of age, all of the combined marker equations show P30≥94.8% in people ≥80 years old. In some cases (BIS2, FAS$_{creatinine\text{-}cystatin}$) this exceeds the accuracy seen in younger individuals (<50 years). In all other cases confidence intervals overlap between these age groups (i.e. not different). In relation to sex, in the majority of cases confidence intervals overlap between males and females. Accuracy does appear lower in males using EKFC$_{creatinine}$ and higher in males using EKFC$_{cystatin}$, with no difference when using the combined EKFC equation. These are exploratory data for which the study was not adequately powered. The limitations of these analyses are reviewed in the discussion (page 13). Ethnicity was not addressed in supplementary Table 3, but in Table 4 there was no significant difference in accuracy between south Asian and white individuals.

5. Role of Albuminuria Not Acknowledged
The manuscript provides a thoughtful reflection on the limits of estimation based on creatinine and cystatin C, noting that biological and analytical variability set an upper limit on achievable accuracy. However, it omits any mention of albuminuria, which is a routinely used complementary marker in CKD diagnosis and risk stratification per KDIGO guidelines. Briefly acknowledging that eGFR is only one part of CKD staging, and that albuminuria provides complementary information, would help contextualise the limitations of the study and ground its findings in routine clinical practice.

We feel we have been clear in the first paragraph of the methods that CKD staging requires knowledge of albuminuria. Our study focussed on the accuracy of GFR equations rather than risk stratification using GFR and albuminuria. We did stratify our data by albuminuria category (supplementary table 3) which, with the exception of the BIS equations (subsequently removed from the paper), did not show a difference in relation to albuminuria.

3. Minor Comments and Editorial Suggestions
In the Supplementary Methods section, the reported P30 value for the BIS1creatinine equation is listed as '94.9 (82.3, 87.3)'. This appears to be a typographical error, as the reported 95% confidence interval does not include the point estimate (94.9), and the lower bound (82.3) is higher than the upper bound (87.3), which is not logically possible. Please review and correct this value to ensure consistency and accuracy of the supplementary data.

Apologies this value should have been 84.9. However, in response to the comments of some other reviewers (see later), we will no longer be including the BIS1 data in our report.

The term 'accuracy' is used frequently, but it would be helpful to define this more precisely in relation to P30, bias, and RMSE at the beginning of the Results or Methods section.

Accuracy was assessed using P30. Accuracy incorporates elements of both bias and precision. We have made this clearer in the methods section (page 10) and elsewhere.

Please ensure that the manuscript is consistent in whether it refers to 'CKD-EPI 2021' or 'new CKD-EPI' and 'original CKD-EPI' versus '2009 CKD-EPI' to avoid confusion.

We apologise for the inconsistency. We have now described the equations as either plain CKD -EPI (meaning the earlier published 2009/2012 versions) and CKD-EPI(2021) in the main body of our report. 'Original' as a descriptor of the equation has been removed. This should improve clarity.

<u>Reviewer 2</u>

This is a companion paper to Scandrett et al, which I apparently read out of sequence. Having reviewed the longitudinal GFR paper, I was asked to review this cross-sectional paper. Several of my overall comments about data presentation are reflected in the other review – specifics to follow. However, whereas I viewed the longitudinal paper as being a highly novel contribution to the literature, this cross-sectional manuscript from the same cohort has less novelty and more limitations in its presentation. The overall design of a 6-center study of measured GFR among persons with CKD remains an excellent achievement. However, several limitations of this cross-sectional study limit its impact and make me question the validity of its conclusions.

We thank the reviewer for his insightful comments, which we have attempted to address as detailed below.

Summary box: the topic summary includes two issues that I found inappropriate.
The relative costs of creatinine and cystatin C. I also practice medicine in a national healthcare system with constrained costs (U.S. Veterans Health), and we are told the actual lab tests for all tests at our hospital. As with this research study, we use enzymatic creatinine, and the cost is $1.50/test. Our cystatin C at moderately high volume is $2.75. For reference, our 25(OH) vitamin D is $10, our PTH is $28, and our NtproBNP is $50. The narrative around the costs of cystatin C is suppressive and beyond that used for any other lab test that I have read literature about. The authors quote relative costs in pounds of 0.43 for Cr (is that enzymatic or Jaffe?) and 3.8 for cysC. Even at this 10-fold quoted cost, nothing about this paper would lead to 50 million cysC tests being done in the UK – the reported number for Cr.

Healthcare costs, the way in which healthcare is funded and laboratory tests requested differs across organisations and countries. Without getting into a comparison of the relative costs of NTpBNP, PTH and vitamin D (which, for example, are very different in the UK from those the reviewer has cited for their organisation), the statement that cystatin C is 'more expensive' than creatinine is true, and it is only reasonable, in our opinion, that more expensive tests should justify their value before implementation. However, we have removed cost as a justification for the study in the abstract objectives and also in the box (key messages).

Subgroups of black and south Asians: the authors were sadly only able to recruit 5% (n=60) each for these two groups. These low numbers warrant noting in the text, but they are far too low for the sweeping conclusions cited in the summary, the abstract, and the discussion. The diverse recruitment for this study was a near-complete failure with about 90% white participants. The fact that diverse recruitment was attempted does not mitigate this fact, and it suggests that the recruited subset may have substantial bias from their representative communities.

We have removed reference to performance in black and Asian individuals from the summary box (key messages) and the abstract and have revised the discussion accordingly. To describe this as a 'near complete failure' is perhaps a little strong. 13% of the population were non-white (Table 1), compared to a population prevalence of 19%. Whilst we had planned to over-recruit individuals from ethnic minority groups, this proved challenging in reality. As we

are sure the reviewer will appreciate, asking individuals to enrol in a 3 year study involving a 4 h infusion procedure at the beginning (and end – in relation to our longitudinal study BMJ-2025-085005), as a minimum, is difficult and studies have repeatedly shown that recruitment from ethnic minority groups can be more challenging (see discussion). Our study was never powered on the number of ethnic minority individuals recruited. To our knowledge, there is currently no larger prospective study including measured GFR of black and Asian subgroups in the UK. Nevertheless, we accept the comment that few conclusions can be drawn from our ethnic minority data.

The need to calibrate cystatin C values: I appreciate the struggle that the authors went through upon learning that the Abbott platform leads to biased cysC values; this is something we learned at the UCSF university hospital in 2020. Calibration is an inexact process. For the longitudinal paper, I think this is less of an issue because changes in the measure are unlikely to be affected by the calibration bias. However, I believe that the authors should re-measure all cystatin C values at baseline for this paper using an optimal method (Siemens perhaps). At 3.8 pounds/test * 1167, this would not have costed too much relative to the massive investment in this study to date. The calibration exercise substantially lowers the confidence an informed reader can have in the results.

The reviewer clearly appreciates the critical importance of calibration in such studies and the efforts we have taken to be both informative and transparent in this respect. By selecting a large number of samples across the required analytical range, demonstrating a close and linear association between the Abbott and Siemens assays (Supplementary Table 1) and Supplementary Figure 2), reanalysis of all samples is unnecessary. The issue was further illustrated by a laboratory recovery study, which has subsequently been repeated (see below). Such recalibration exercises are not uncommon in long-term research studies (see Karger et al Clinical Chemistry 2021 425-433).

Choice of a cohort with eGFRcr 30-60: This seemed appropriate for the longitudinal paper, as these are the patients who require monitoring. However, this creates biases for the study that make Cr look both worse and better. Although mentioned in the discussion, the bias is substantial. A) Unfair to Cr – the results state that Cr has more bias at the low and high values of mGFR; this is self-evident because mGFR can range below and above the 30-60 interval and eGFRcr cannot. This is a study limitation not a research finding. B) Cr looks better – by definition, all participants have recognized CKD, and 60% have substantial urine albumin suggesting they may be recruited from nephrology clinic. Creatinine's major weakness is its insensitivity which increases with age and frailty. So, Cr has a relative advantage in this study that is a major bias compared with an unselected population.

The choice of cohort (patients with CKD stage 3 [GFR 30-60 mL/min/1.73 m^2]) was defined by the National Institute of Health Research when commissioning the study. Such patients represent the majority of patients with CKD and low excretory function and are therefore an important group in whom assessment of the accuracy of GFR equations should be studied. We have mentioned the restriction of our cohort to such individuals as a study limitation in the discussion, although in practice 26% of patients had GFR measurements outside this range. The reviewer seems to be suggesting that this is biasing the study both against creatinine ('unfair to creatinine') and in favour of creatinine ('Cr looks better'). We do not fully follow this argument. The median age of our cohort was 67.5 years, with 25% of participants >74.5 years old. There was extensive co-morbidity.

Results presentation:
Figure 1 – I found these 15 panels to be unreadable. Maybe 2 panels would work or spread across multiple pages.

We accept this point, which is also supported by comments from reviewers 5 and 6. In combination with the following comment from this reviewer (and comments relating to our longitudinal study BMJ-2025-085005) we have now limited our data presentation to the CKD-EPI (2009/2012) and EKFC equations. The manuscript has been revised accordingly. Figure 1 will therefore have 6 panels. The equivalent figures for the two CKD-EPI(2021) equations have been placed in the supplementary file, as we feel there may still be considerable interest in the relative performance of these.

Table 2 – a) as in my review of the other paper, the main findings should show only 6 equations – Cr, cys, both for 2 equation families. The race or no-race issue cannot be decided in this study given 60 black persons. All other equations can be parked in a supplement.

We agree (as above). The black/south-Asian data has been downgraded in importance (although it was already heavily caveated) and pointers will be given to the availability of the other equations data.

Table 3 – The key findings here are that the combined equations are all 5% higher on the P30 scale compared with the Cr referent equation. I still believe a figure could summarize these results, limiting to the top 6 equations with others in supplement.

Table 3 has been moved to the supplementary file (Supplementary Table 2) and now only includes the CKD-EPI and EKFC equations, which simplifies the presentation. Significant positive comparisons have been highlighted in bold. The 5% improvement in P30 of the combined equations is also readily apparent from Table 2.

Table 4 – these data have the small black and south Asian groups. Its fine to show the data, but these are basically pilot data. A statement was made about significance for the Black subgroup with race-free equation; this did not seem appropriate as based on the white distribution and not compared with the paired results from the data with race included. The absolute difference of 5% is the powered minimum detectable difference for the entire cohort of 1000+.

We wonder if there has been a misunderstanding here. The p-values shown are for the P30 black subgroup (n=60) with and without the race factor e.g. 81.7% compared to 70.0% for CKD-EPIcreatinine. However, this only achieved significance for the MDRD equation, which has now been removed from the paper, and Table 4 has been moved to the supplementary file (Supplementary Table 4).

Supplementary Table 3 – this table is overly dense, but these data on varied performance by subgroups of age, sex, are very important and could be presented in the main paper in a condense format. Again, figures are efficient for overviews of lots of data.

Having removed many of the studied equations, this table is less dense. However, the study was not powered for these analyses and there are limitations to these data (see discussion page 13). We would prefer to leave this as a table in the supplementary file.

Discussion – As already stated I think the conclusion about race subgroups are inappropriately based on very small numbers. For the South Asian subset, for example, it might be informative to cite a

paper by my colleagues from the UK Biobank among 7,738 South Asians, albeit without mGFR (Chen DC JAHA, 2023) https://pubmed.ncbi.nlm.nih.gov/36695320/.

Thank you for pointing us towards this interesting paper, adding to a significant body of literature detailing the improved value of cystatin C as a marker of risk compared to creatinine. However, our study concerned accuracy of GFR estimates compared to measured GFR, which was not addressed by Chen et al.

The authors make important points about the challenges of an imprecise reference standard, mGFR, and the limited ability of biomarkers to exceed the precisions described in this paper.

Thank you

Regarding cysC laboratory methods, the authors cite studies from 2017, whereas papers by Amy Karger suggest that the measurement and standardization are improving.

We would hope that standardisation of cystatin C assays is improving. However, when we repeated our recovery exercise in 2024 we found an over-recovery in the Abbott assay of 9.3%, compared to 12.4% when we first did this analysis in 2018. This would suggest that the assay is still over-recovering. This is described in the supplementary methods section. Thank you for pointing us towards the paper by Karger et al (Clinical Chemistry 2021 425-433) This is of interest, but in fact does still describe some bias and drift in their cystatin C measurements over time and beyond 2017 (their Figure 2), even when plotted against +/- 3SD limits (2 SD limits are generally utilised in laboratory medicine). The impact of recalibration is also dramatically illustrated in their Figure 1. It should also be noted that this paper, and a later paper from the same group (Karger A et al. Improved Performance in Measurement of Serum Cystatin C by Laboratories Participating in the CAP 2019 CYS Survey. Arch Pathol Lab Med. 2022), does not address the Abbott assay. The issue of cystatin C standardisation is clearly not fully resolved and remains a challenge to studies of equations utilising cystatin C, particularly long-term studies (as Karger et al acknowledge). For the reviewers information, Abbott acknowledged in 2018 that during the period of our baseline measurements (2012-2018) there had been an 8% upward drift in their calibration, but this remained within their +/- 5% manufacturing tolerance. Until manufacturers better understand the clinical and research impact of 'drift' in their products, this is likely to be an ongoing issue which we feel is important to highlight.

Reviewer 3
Comments: None

Reviewer 4

Thank you for the opportunity to provide review for this well-written paper.
Originality - does the work add enough to what is already in the published literature? If so, what does it add?
The article assess the accuracy of GFR estimating equations, including those utilizing cystatin C, in a representative English population comprising adults with stage 3 CKD (MDRD creatinine- estimated GFR of 30-59 mL/min/1.73 m2, sustained for at least 3 months prior to recruitment). The study includes black and South Asian individuals from six UK centers. At present, there are no large-scale multi-center studies comparing estimating equations with measured GFR. The article is innovative and provides high-level evidence for addressing urgent problems in current clinical practice.

Importance of work to readers - does this work matter to researchers, clinicians, patients, teachers, or policymakers?
The staging of CKD is primarily based on GFR. Measured GFR values are impractical for routine clinical use and GFR is typically estimated using established equations. Different CKD stages require distinct treatment strategies. An inaccurate GFR estimation can lead to completely different treatment measures, and result in poor prognoses for patients near a stage threshold. Therefore, an accurate GFR estimation through validated equations is crucial for precise CKD staging. The findings in this article have significant implications for researchers, clinicians, patients, teachers and policymakers alike.
Evidence-based medicine - is the evidence presented scientifically and reliable?
The research data were obtained from six centers, featuring a reasonable sample size, an acceptable dropout rate, and a well-balanced baseline distribution. The statistical analysis methods were appropriate, and the evidence presented were both scientifically valid and reliable.
Research question - is it clearly defined and appropriately answered?
It was clearly defined and appropriately answered.
Study design - is the overall design adequate?
The overall design of study was appropriate and adequate to answer the research question.
Participants - are they adequately described and their conditions well defined?
In the supplementary file(P32), participants were adequately described, the conditions and both interventions were clearly defined, inclusion and exclusion criteria were explicitly stated and a rationale was given for these decisions.
Methods - are they adequately described? Are they ethical?
The methodology was described in this article and previously published protocol, enabling readers to access comprehensive information. This study adhered to the fundamental principles of ethics.
Results - do they answer the research question? Are they credible and well-presented?
The results had answered the research question, demonstrating credibility and clarity in presentation. However, considering the topic's popularity and its potential to attract significant interest, minor revisions are needed to enhance expression. For instance, in Supplementary Figure 2, the Gantt chart, although the article has provided an explanation for the dropout cases, it would be beneficial to add arrow lines connecting the dropped-out subjects to the corresponding stages to make the logic becomes clearer.

Thank you for pointing this out. We have added lines to the study flow chart in Supplementary Figure 1.

Interpretation and conclusions - are they warranted by and sufficiently derived from/focused on the data? Is the message clear?
These conclusions were warranted by and sufficiently derived from/focused on the data. There were no distractions or irrelevant conclusions and the findings were discussed in the light of previous evidence. The study was elegantly presented and the message was clear. In particular, the author intends to analyze potential differences in estimated GFR using equations among different races, especially the black population. In the results section, 60 black individuals were actually enrolled. The author compared number of black cases obtained in this study with the proportion of the black population in the UK, and analyzed the results objectively.
I recommend that the author revise the discussion section. Analyze and discuss in the order of :
1.All GFR estimates showed negative bias overall, with creatinine-based equations exhibiting a particularly pronounced shift in bias depending on level of GFR compared to reference measured GFR.
2.The inclusion of cystatin C within combined equations reduced bias overall and lessened, though did not entirely eliminate, the bias/GFR level effect. Several equations, especially the combined

creatinine and cystatin equations, achieved clinically acceptable performance as defined by P30>90%.
3.Simple removing the black race adjustment factor from creatinine-based GFR equations may reduce accuracy.
The discussion should clear and concise, and avoid interweaving the analyses.

Thank you. The discussion has been extensively revised taking these comments into account where appropriate.

References - are they up to date and relevant? Any omissions?
Most of the references were current, appropriate for the content and highly relevant. While some references were not up to date, they remained representative and thus none of the references required omission.
Abstract/summary/key messages - do they reflect accurately what the paper says?
I recommend the "Abstract" needs to be revised.
1.The "Objective" should be revised to, for example: To compare the diagnosis validity of estimates of GFR equations to measured GFR.

Objective revised

2.The "Design" should be revised to: prospective, multi-center.

Revised

3.The "Participants" should be revised to: adults with moderate CKD (estimated GFR 30-59 mL/min/1.73 m2 sustained over at least 3 months prior to recruitment).

Revised

4.The "Summary" need no revision.
Reporting standards - does the paper comply with relevant standards? If the paper is a randomized controlled trial we will have asked the authors to provide the protocol and a CONSORT checklist. Other research designs should have the relevant checklist included (PRISMA, STARD, etc).
The article is not a randomized experiment and complies with relevant standards.

<u>Reviewer 5</u>

In this study of patients with stage III chronic kidney disease (CKD) from six primary, secondary, and tertiary care centers in England, Lamb and colleagues evaluated the accuracy and precision of 15 published equations for estimating glomerular filtration rate (GFR). A total of 1,167 adults with confirmed stage III CKD were included. Estimated GFR values were compared against measured GFR using iohexol clearance. The study has been registered and there is a considerable overlap with a publication in has been published in the National Institute of Health Research Journals Library. Accuracy was assessed using the P30 metric (percentage of estimates within 30% of the reference value), as recommended by the National Kidney Foundation–Kidney Disease Outcomes Quality Initiative. The findings showed a critical role of assay calibration for cystatin C measurements; following calibration adjustment, all equations achieved a P30 above 84.1%. Creatinine-based equations demonstrated variable bias depending on GFR level, while equations combining creatinine

and cystatin C yielded higher accuracy than those based on a single biomarker. Differences in performance across subgroups defined by age, sex, diabetes status, albuminuria, body mass index, GFR, and ethnicity were equation-specific, with notably reduced accuracy among Black individuals when the race coefficient was omitted.

Given the widespread use of GFR estimating equations in clinical practice, this study offers valuable insights not only for general practitioners. The methodology is clearly described and appropriately executed, and the results are presented in a clear and accessible manner. Nonetheless, several study aspects could benefit from further clarification or refinement. The authors may want to consider the following aspects.

1. From both the main manuscript and the supplementary information, it remains unclear what the target population is and how the study sample reflects that population. One may infer that the target population comprises individuals with stage III CKD living in the catchment areas of the six participating centers (Birmingham, Canterbury, Derby, Leicester, Salford, and London [King's College Hospital]). However, it is not specified how many individuals with stage III CKD reside in these areas, how many were seen at the centers, how many were invited to participate, or what proportion ultimately enrolled. The flow chart shown in Supplementary Figure 2 does not help clarify these points. As such, the generalizability of the study findings remains uncertain.

Reliable CKD3 prevalence data from each of the centres is not available. There is published data from the Canterbury area where a conservative estimate of population prevalence is 4.0%, and from Leicestershire, where the CKD3 population was estimated to be 5.9%. We have added this information to the first paragraph of the introduction. There is information in the supplementary file describing the number invited, the number participating and the number with evaluable data (Supplementary file pages 1-2).

2. Related to my previous point, I recommend that the authors refrain from describing the sample as "representative." It is unclear whether the participants constitute a representative sample of the catchment area populations or patients with stage III CKD more broadly. Moreover, the proportion of non-white participants was approximately 31% lower than reported in recent census data, a difference I would not consider "slight." It is also unclear whether the census figures refer specifically to the populations served by the six participating centers. This further limits claims of representativeness. The lower proportion of non-white participants may also reduce the statistical power for subgroup analyses within this population.

We have removed reference to the study population as representative. We have removed the statement that non-white participation was 'slightly' below census data. The census data is national and we think this is clear from the cited publication. There is undoubtedly variation between the centres in terms of non-white population prevalence as would be expected. Throughout the manuscript we have further caveated any conclusions we may draw regarding the non-white data, as also suggested by reviewer 2.

3. The authors should report the time frame during which participants were recruited. This information is difficult to find, even when looking the registered protocol or the study's website. The study website indicates that 1,249 participants were recruited over a 35-month period, which differs from the 1,229 participants reported in the submitted manuscript. This discrepancy should be clarified.

The timeframe over which recruitment occurred is clearly stated in the supplementary file (page 1, April 2014 to January 2017). The correct number of participants is 1,229. Thank you for highlighting this discrepancy. The study website has now been corrected.

4. The authors should already state in the Introduction of the Methods section what the rationale was to use the P30 cut point. If I am not mistaken, this is only mentioned in the supplementary material.

The methods section in the main paper clarifies what P30 is (i.e. the proportion of GFR estimates within 30% of measured GFR) and cites the NKF as the source of the recommendation for this approach.

5. The details in Figure 1 are difficult to interpret, even when zooming in to the maximum extent. The 15 individual panels within the figure are too small to be readable. I also recommend avoiding using red and green as contrasting colors (in all Figures), which may hinder interpretation for individuals with red-green color vision deficiency.

We agree and other reviewers have also commented on this. In the revised manuscript we have reduced the number of equations to the CKD-EPI and EKFC equations only, so the figure now only has 6 panels. We have also changed the colour scheme.

6. The authors should consider providing a rationale for selecting the covariates used for stratification in the explanatory analyses. Additionally, some continuous variables were dichotomized, which may obscure relevant information.
The covariates considered in the exploratory analyses are fairly standard and reasonable to include within this field. The stratification points are also fairly self-evident and supported by references where appropriate (e.g. NICE stratification of BMI). We have added an explanation of the choice of 45 mL/min/1.73 m^2 as the GFR threshold. These were exploratory analyses and their limitations have been addressed in the discussion (page 13).

7. The titles of the Tables are pretty long. The authors may consider moving some information into the corresponding table footnotes to improve readability.

Thank you for this suggestion. We have moved material to footnotes accordingly where appropriate.

8. I do not see a conflict of interest, but the authors may want to reconsider the statement that support from pharmaceutical companies was unrelated to the study. Several of these companies provide diagnostic materials or medications used in managing CKD, and given that the study addresses diagnostic accuracy, this relationship may still be relevant.

We are unaware of diagnostic products relevant to this field manufactured and marketed by the named pharmaceutical organisations.

<u>Reviewer 6</u>

The authors evaluate eGFR accuracy in adults patients with CKD, using 15 different published equations. The conclusion that the CKD-EPI formula with creatinine performs reasonably well (with measured iohexol clearance as ground truth), and that incorporating cystatin C has added value in this context, is relevant. The prospective study was performed in 1167 individuals recruited at 6 hospitals in the UK. Overall, this appears to be a solid and well-conducted study.

General Comments

- One could question the representativeness of the findings for clinical practice, as the study was conducted in what seems to be an idealized setting. I suspect that in real-world clinical environments, the differences between the equations may be even smaller.

We have discussed this issue on page 14 of the manuscript. In clinical practice, creatinine and cystatin C would be measured in a variety of laboratories, increasing the between-centre imprecision of the results with onward impact on GFR estimates. This is likely to decrease the agreement between estimated and measured GFR (lower P30 values). Whether it would also reduce relative differences in accuracy between equations seems less predictable.

- The manuscript is somewhat difficult to follow due to a lack of clear structure and limited visual support. A more intuitive narrative and better use of figures would greatly enhance readability. Figure 1, for example, is currently unreadable and conceptually unclear. Moreover, plotting bias versus measured GFR is less intuitive than plotting estimated versus measured GFR, which would allow for a more direct assessment of accuracy. Including R^2 values in such plots would also help readers better understand the performance of the equations.

We have extensively revised the manuscript and hope that is now more structured and readable. We agree regarding Figure 1 and this has been reduced to a 6-panel figure. There are good arguments for presenting data such as this as difference plots rather than agreement (correlation/scatter) plots and we would prefer to retain this style. We have now included R^2 values in Table 2 as an additional aid to understanding equation performance.

- Table 2 would benefit from visualization to make the data more accessible.

Thank you for this suggestion. We agree and have now included an additional box and whisker figure to illustrate this data (new Figure 1).

- Table 3 is dense with p-values, making it difficult to identify the key findings. While the differences in P30 scores are not incorrect, they are somewhat abstract and hard to interpret without additional context.

Table 3 has been reduced to show only the CKD-EPI and EKFC equations which simplifies the presentation. We still feel that the table provides important objective comparison between the equations but have moved the table to the supplementary file (now supplementary Table 2). The key findings are now shown as bold p-values.

- The use of P30 as the primary accuracy metric stands out. While this is in line with NKF and KDIGO recommendations, it remains a somewhat arbitrary threshold. It is commendable that the authors also report RMSE in Table 2, which provides a more continuous measure of error. I would strongly recommend also including R^2 values and corresponding visualizations to complement the P30 and RMSE metrics.

We agree that the P30 value is not an ideal metric and have referenced this in our Discussion (page 13). However, it is almost universally used in the GFR research field and its inclusion allows comparison between studies. We have added R^2 values to Table 2.

- The limited representation of non-White individuals is a constraint, though this is acknowledged and

appears to be a limitation of the cohort itself. The overall good performance of all equations may be partly due to the CKD stage 3 population. It is unclear why the recruitment was not extended to a broader population, which could have improved the generalizability of the findings.

The choice of cohort (patients with CKD stage 3 [GFR 30-60 mL/min/1.73 m^2]) was defined by the National Institute of Health Research when commissioning the study. Such patients represent the majority of patients with CKD and low excretory function and are therefore an important group in whom assessment of the accuracy of GFR equations should be studied. We have mentioned the restriction of our cohort to such individuals as a study limitation in the discussion, although in practice 26% of patients had GFR measurements outside this range.

Overall, this is a well-executed study that contributes to the growing literature on the best choice of eGFR formula. My comments above hopefully help improve the manuscript, especially for a general readership.

Thank you

VERSION 2 – REVIEW

**REVIEWER NAME**	Ikenoue, Tatsuyoshi
**REVIEWER AFFILIATION**	
**REVIEWER CONFLICT OF INTEREST**	None
**DATE REVIEW RETURNED**	18-Jul-2025

**GENERAL COMMENTS**	My question has been answered.I hope it will be a good paper

**REVIEWER NAME**	Shlipak, Michael
**REVIEWER AFFILIATION**	University of California, San Francisco
**REVIEWER CONFLICT OF INTEREST**	None
**DATE REVIEW RETURNED**	28-Jul-2025

**GENERAL COMMENTS**	Overall, this manuscript has been substantially improved through revision, and the results seem appropriately discussed and presented. With this overall, highly favorable view to the revision, my following comments are:<br><br>The ABBOTT assay. At the ADLM conference this week, I have learned that the Abbott platform and Abbott reagents are not necessarily the same thing. In the U.S., Abbott now uses a different company's assay on its platform. So, this distinction might be helpful for an audience that may not understand the distinction.<br>The slope of 0.94 in the calibration between the disgraced Abbott assay and the Siemens is not as high as it should be. The slope of correlation between two top products, Siemens and Gentian, was 0.96 in a paper I was involved in (https://pubmed.ncbi.nlm.nih.gov/21310869/) . Therefore, mere calibration does not eliminate the excess error from the Abbott

	assay. This should be noted in the discussion that the eGFRcys and eGFRcr-cys equations may have under-performed as a result of the flawed assay – despite calibration.<br>Related to the eGFRcr range of 30-60, the authors need to clarify that their study cannot study persons with unrecognized CKD due to eGFRcr > 60, whereas the mGFR or eGFRcr-cys might signal CKD.<br>Another topic that was inadequately addressed are the R squared and RMSE data added to Table 2 at request of another reviewer. Why do the Cr alone equations look so much worse based on these statistics in comparison with the other equations that include cysC? I think this needs to be discussed. Even though the P30 is generally accepted as the primary metric in mGFR work, this measure was devised by the eGFRcr developing group in hopes of providing confidence to the clinical community. It might not be ideal for distinguishing between these equations in this cohort.<br>These are my major points, although I do agree with the key messages drafted by the authors and with the remainder of the discussion section.

**REVIEWER NAME**	Davies, Neil
**REVIEWER AFFILIATION**	UCL, Division of Psychiatry and Department of Statistical Science
**REVIEWER CONFLICT OF INTEREST**	None
**DATE REVIEW RETURNED**	26-Aug-2025

**GENERAL COMMENTS**	bmjmed-2025-001827 Glomerular filtration rate estimation using creatinine and cystatin C: a prospective study of test accuracy in adults with moderate chronic kidney disease<br><br>This is an interesting study that investigated the accuracy of a range of models for kidney function versus a gold standard GFR. The study is well conducted, and I have no major comments about the analysis. However the results are complex and inconclusive and not particularly clearly presented. For example, it is not clear which of the models is the current standard practice, and which reflect additional measures.<br><br>The figures are not particularly informative. For example, it is not clear which model (if any) is better from the Box and Whiskers plot in Figure 1. Figure 2 presents the bias of the different estimating methods, but again it is not clear what inference can be drawn, beyond all methods are slightly biased. This was noted by the reviewers in the first version of the paper, and the revised paper has not improved this.<br><br>Table 2 presents the estimated accuracy of the different estimation methods. However, it is quite hard to interpret. First, it would be helpful if the sensitivity analyses using the assay without recalibration was relegated to the appendix. This is distracting for your readers without adding much useful additional information. Throughout, the key message from this table is not clearly articulated, which method is best? Or are they are all pretty similar? The R2 and RMSE suggest quite substantial differences, but the key message as set by the parameter set by the NKF (P30), suggests that all dual biomarker estimates achieved the benchmark and CKD-EPIcreatinine and EKFCcystatin.<br><br>The supplementary materials investigates whether there is any

	heterogeneity in the predictive accuracy of these tests in different ethnicities. However, these samples are far too small to accurately test this hypotheses.<br><br>As noted by the reviewers, there is no reason to presume that this sample is representative of the population, which may limit generalisability.<br><br>The overall conclusions, that original CKD-EPIcreatinine equation has acceptable accuracy is not particularly compelling.<br><br>Minor comments<br>"Supplementary Table 3 presents accuracy of GFR estimating equations by categories of<br>age, sex, diabetes status, albuminuria, BMI and level of GFR. There was evidence of<br>differences in accuracy in relation to certain characteristics, as indicated by non-overlapping 95% CIs." It would be worth formally testing for differences across each of these characteristics and including a summary of this test clearly in each table. Currently readers have to eye-ball the supplementary table and qualitatively assess heterogeneity across groups.<br><br>It would be worth considering whether the TRIPOD checklist or other similar checklist for predictive models could be used and reported.<br><br>It would be helpful if the code used to clean and analyse the data could be shared on a repo like GitHub etc or as a supplement.

VERSION 2 – AUTHOR RESPONSE

Reviewer: 1

My question has been answered. I hope it will be a good paper

Thank you

Reviewer: 2

Overall, this manuscript has been substantially improved through revision, and the results seem appropriately discussed and presented. With this overall, highly favorable view to the revision, my following comments are:

1. The ABBOTT assay. At the ADLM conference this week, I have learned that the Abbott platform and Abbott reagents are not necessarily the same thing. In the U.S., Abbott now uses a different company's assay on its platform. So, this distinction might be helpful for an audience that may not understand the distinction.

Our study was undertaken using Abbott reagents on an Abbott Architect analyser. We are aware that Abbott are providing a new kit for use on the Abbott Alinity analysers, although this uses reagents from another company (Sentinel). This may be what the reviewer is referring to. This new kit version is not currently available in the UK. Actually the original Abbott Architect application also used Sentinel reagents, but badged as Abbott. Accuracy of all assays may

change over time and we feel that further discussion of this issue would probably not be helpful for the audience. We repeated our recovery exercise using international standard cystatin C in 2024 and obtained essentially the same result as that achieved in 2018. This information is in the supplementary material.

2. The slope of 0.94 in the calibration between the disgraced Abbott assay and the Siemens is not as high as it should be. The slope of correlation between two top products, Siemens and Gentian, was 0.96 in a paper I was involved in (https://pubmed.ncbi.nlm.nih.gov/21310869/) . Therefore, mere calibration does not eliminate the excess error from the Abbott assay. This should be noted in the discussion that the eGFRcys and eGFRcr-cys equations may have under-performed as a result of the flawed assay – despite calibration.

We believe that the value of 0.96 the reviewer is referring to is a correlation coefficient, whereas the value of 0.94 in our paper is the slope describing the relationship between the two methods in a regression equation. (The Pearson's correlation coefficient for the agreement between the Abbott and Siemens methods was actually 0.994 – this is now stated in the Results section of the paper, page 11). We used two different regression approaches (linear and Deming) to obtain the equation to recalibrate cystatin C, and both gave similar results, as described in the supplementary file. These regression equations were not intended to be the definitive approach to recalibration, but to illustrate the scale of the difference that inaccurate calibration can induce in cystatin C-based GFR estimates. However, we agree that the error in the Abbott assay is probably not just a calibration issue. This has been discussed in the supplementary file (penultimate paragraph).

3. Related to the eGFRcr range of 30-60, the authors need to clarify that their study cannot study persons with unrecognized CKD due to eGFRcr > 60, whereas the mGFR or eGFRcr-cys might signal CKD.

We are unclear of the point being made here. We have already stated that any conclusions relating to people with GFR outside the study target recruitment range are limited.

4. Another topic that was inadequately addressed are the R squared and RMSE data added to Table 2 at request of another reviewer. Why do the Cr alone equations look so much worse based on these statistics in comparison with the other equations that include cysC?

I think this needs to be discussed. Even though the P30 is generally accepted as the primary metric in mGFR work, this measure was devised by the eGFRcr developing group in hopes of providing confidence to the clinical community. It might not be ideal for distinguishing between these equations in this cohort.

Thank you, we have now included the following in the results: "The R2 values range from 0.53 to 0.71 showing moderate positive linear association. RMSE values range from 7.06 to 8.99 indicating the scale of potential error with which estimating equations approximate measured GFR. Use of cystatin C, particularly in the combined equations, tended to improve precision and, for the EKFC equations, reduced bias compared to measured GFR (Figure 1)."

5. These are my major points, although I do agree with the key messages drafted by the authors and with the remainder of the discussion section.

Thank you.

Reviewer: 3.

6. This is an interesting study that investigated the accuracy of a range of models for kidney function versus a gold standard GFR. The study is well conducted, and I have no major comments about the analysis. However the results are complex and inconclusive and not particularly clearly presented. For example, it is not clear which of the models is the current standard practice, and which reflect additional measures.

The introduction (second paragraph) contains a statement that CKD-EPIcreatinine is currently recommended for clinical use. We have added a sentence to the objectives section of the abstract to further clarify that creatinine-based estimates of GFR represent current clinical practice in many countries (as also suggested by the Editor – see below, point #17).

7. The figures are not particularly informative. For example, it is not clear which model (if any) is better from the Box and Whiskers plot in Figure 1. Figure 2 presents the bias of the different estimating methods, but again it is not clear what inference can be drawn, beyond all methods are slightly biased. This was noted by the reviewers in the first version of the paper, and the revised paper has not improved this.

Figure 1 (Box and Whiskers plot) was included at the specific request of reviewer 6 (MN) in relation to the earlier version of the manuscript. The reviewer correctly interprets that most estimates show a slight negative bias compared to measured GFR, as stated in our discussion (second sentence). The figure illustrates that the use of combined equations reduces the spread of data and, in the case of EKFCcreatinine-cystatin, the bias compared to creatinine-only equations. Some of this information can also be obtained from Table 2, but we feel that the figure also better illuminates some aspects e.g. the reduced spread of the central two quartiles with the combined equations.

Figure 2 (lowess plots) included 15 panels (equations) in an earlier version of the manuscript and several reviewers of that version criticised the figure (justly) for its complexity rather than its relevance. The figure illustrates an important point that the bias of GFR equations is not constant across all levels of kidney function (measured GFR), and that the inclusion of cystatin C ameliorates this effect. This is mentioned in the abstract results, the main results and the discussion.

8. Table 2 presents the estimated accuracy of the different estimation methods. However, it is quite hard to interpret. First, it would be helpful if the sensitivity analyses using the assay without recalibration was relegated to the appendix. This is distracting for your readers without adding much useful additional information. Throughout, the key message from this table is not clearly articulated, which method is best? Or are they are all pretty similar? The R2 and RMSE suggest quite substantial differences, but the key message as set by the parameter set by the NKF (P30), suggests that all dual biomarker estimates achieved the benchmark and CKD-EPIcreatinine and EKFCcystatin.

We have moved the pre-recalibration data to the Supplementary file (Supplementary Table 2) and referred to this in a footnote to Table 2. Some supporting narrative to this table has been modified in the Results section. We agree that this now clearer. (see also response #4 above).

As you would expect, those equations achieving the highest P30 values also tended to have higher R2 and lower RMSE and bias values. The P30 metric is used as it incorporates elements of both accuracy and bias. In terms of which equation is 'best', the data in supplementary Table 3 clearly show that the combined equations are statistically superior in terms of P30. Other issues that one would consider when choosing an equation is whether it is unduly influenced by other characteristics, for example those considered in Supplementary Table 4, and consistency of bias across the GFR range (Figure 1). All of these issues have been considered in our study and detailed in the discussion. Our data cannot discriminate between the CKD-EPI and EKFC combined equations, but what we can discern is that both combined equations are generally superior to their single biomarker equivalents. This is consistent with our final conclusion in the key messages, main paper and the abstract.

9. The supplementary materials investigates whether there is any heterogeneity in the predictive accuracy of these tests in different ethnicities. However, these samples are far too small to accurately test this hypotheses.

This is exploratory data but we make it extremely clear in our discussion that the study was underpowered to answer this question. However, we feel that it would be a mistake to completely ignore these observations, which could have significant impact, and hope that this will stimulate further research addressing this issue.

10. As noted by the reviewers, there is no reason to presume that this sample is representative of the population, which may limit generalisability.

Whilst we did not adopt a formal random selection approach to ensure that the study population was representative of the overall population, the data indicate that the study population is similar with respect to demographic and comorbidity variables, to other cohort studies from the UK. CKD is intrinsically highly variable and it is therefore challenging to make a cohort representative of all people with CKD. We therefore focussed on CKD category 3, the most prevalent diagnosed category. We have added a statement regarding generalisability to the limitations section.

11. The overall conclusions, that original CKD-EPIcreatinine equation has acceptable accuracy is not particularly compelling.

We would agree, but that is the case. As we describe in the discussion P30>90% is not a particularly high bar. However, the overall conclusion also goes on to state that combined creatinine-cystatin containing equations can improve on this situation, which is an important conclusion.

Minor comments

12. “Supplementary Table 3 presents accuracy of GFR estimating equations by categories of age, sex, diabetes status, albuminuria, BMI and level of GFR. There was evidence of differences in accuracy in relation to certain characteristics, as indicated by non-overlapping 95% CIs.” It would be worth formally testing for differences across each of these characteristics and including a summary of this test clearly in each table. Currently readers have to eye-ball the supplementary table and qualitatively assess heterogeneity across groups.

We always planned for this analysis to be descriptive and we are not powered to formally look at differences between these groups. The sample size for some is too small to offer meaningful results. Our preference is for these to remain as descriptive analyses but we will add these into the paper at the request of the Editor.

13. It would be worth considering whether the TRIPOD checklist or other similar checklist for predictive models could be used and reported.

The TRIPOD checklist is for the development and validation of prediction models. Our study is not concerned with the development of new prediction models. The GFR-estimating equations have been previously developed by others. We feel that the STARD checklist that we have provided is more appropriate for our study and, indeed, already has considerable overlap with many of the criteria of the TRIPOD checklist.

14. It would be helpful if the code used to clean and analyse the data could be shared on a repo like GitHub etc or as a supplement.

An anonymised data set will be available on the University of Birmingham eData Repository at https://edata.bham.ac.uk/. The statistical and Birmingham Clinical Trials Unit teams are currently preparing this and it will be available after 7th November.
